# Computing a maximum clique in geometric superclasses of disk graphs

**DOI:** 10.1007/s10878-022-00853-2

**Published:** 2022-03-25

**Authors:** Nicolas Grelier

**Affiliations:** Department of Computer Science, ETH, Zürich, Switzerland

**Keywords:** Pseudo-disks, Line transversals, Intersection graphs

## Abstract

In the 90’s Clark, Colbourn and Johnson wrote a seminal paper where they proved that maximum clique can be solved in polynomial time in unit disk graphs. Since then, the complexity of maximum clique in intersection graphs of *d*-dimensional (unit) balls has been investigated. For ball graphs, the problem is NP-hard, as shown by Bonamy et al. (FOCS ’18). They also gave an efficient polynomial time approximation scheme (EPTAS) for disk graphs. However, the complexity of maximum clique in this setting remains unknown. In this paper, we show the existence of a polynomial time algorithm for a geometric superclass of unit disk graphs. Moreover, we give partial results toward obtaining an EPTAS for intersection graphs of convex pseudo-disks.

## Introduction

In an *intersection graph*, every vertex can be represented as a set, such that two vertices are adjacent if and only if the corresponding sets intersect. In most settings, those sets are geometric objects, lying in a Euclidean space of dimension *d*. Due to their interesting structural properties, the intersection graphs of *d*-dimensional balls, called *d*-ball graphs, have been extensively studied. For dimensions 1, 2 and 3, the *d*-ball graphs are called *interval graphs*, *disk graphs* and *ball graphs*, respectively. If all *d*-balls have the same radius, their intersection graphs are referred to as *unit d-ball graphs*. The study of these classes has many applications ranging from resource allocation to telecommunications (Bar-Noy et al. 2001; van Leeuwen 2009; Fishkin 2003).

Many problems that are NP-hard for general graphs remain NP-hard for *d*-ball graphs, with fixed $d\ge 2$. Even for unit disk graphs, most problems are still NP-hard. A famous exception to this rule is the problem of computing a maximum clique, which can be done in polynomial time in unit disk graphs as proved by Clark et al. (1990). Their algorithm requires the position of the unit disks to be given, but a robust version of their algorithm, which does not require this condition, was found by Raghavan and Spinrad (2001). This is a nontrivial matter as Kang and Müller have shown that the recognition of unit *d*-ball graphs is NP-hard, and even $\exists {\mathbb {R}}$-hard, for any fixed $d\ge 2$ (Kang and Müller 2012).

Finding the complexity of computing a maximum clique in general disk graphs (with arbitrary radii) is a longstanding open problem. However in 2017, Bonnet *et al.*, found a subexponential algorithm and a quasi polynomial time approximation scheme (QPTAS) for maximum clique in disk graphs (Bonnet et al. 2018). The following year, Bonamy et al. (2018) extended the result to unit ball graphs, and gave a randomised EPTAS for both settings. The current state-of-the-art about the complexity of computing a maximum clique in *d*-ball graphs is summarised in Table 1.

**Table 1:** Complexity of computing a maximum clique in *d*-ball graphs

	Unit *d*-Ball graphs	General *d*-ball graphs
$d=1$	Polynomial (Gupta et al. 1982)	Polynomial (Gupta et al. 1982)
$d=2$	Polynomial (Clark et al. 1990)	Unknown but EPTAS (Bonnet et al. 2018; Bonamy et al. 2018)
$d=3$	Unknown but EPTAS (Bonamy et al. 2018)	NP-hard (Bonamy et al. 2018)
$d=4$	NP-hard (Bonamy et al. 2018)	NP-hard (Bonamy et al. 2018)

Bonamy et al. show that the existence of an EPTAS is implied by the following fact: For any graph *G* that is a disk graph or a unit ball graph, the disjoint union of two odd cycles is a forbidden induced subgraph in the complement of *G*. Surprisingly, the proofs for disk graphs on one hand and unit ball graphs on the other hand are not related. Bonamy et al. ask whether there is a natural explanation of this common property. They say that such an explanation could be to show the existence of a geometric superclass of disk graphs and unit ball graphs, for which there exists an EPTAS for solving maximum clique.

By looking at Table 1, a pattern seems to emerge: The complexity of computing a maximum clique in $(d-1)$-ball graphs and unit *d*-ball graphs might be related. We extend the question of Bonamy *et al.* and ask for a class of geometric intersection graphs that (1) contains all interval graphs and all unit disk graphs, and (2) for which maximum clique can be solved in polynomial time. Recall that the complexity of maximum clique in disk graphs is still open. Therefore a second motivation for our question is that showing the existence of polynomial time algorithms for geometric superclasses of unit disk graphs may help to determine the complexity of maximum clique in disk graphs.

We introduce a class *C* of geometric intersection graphs which contains all interval graphs and all unit disk graphs, and show that maximum clique can be solved efficiently in *C*. Furthermore, the definition of our class generalises to any dimension, i.e. for any fixed $d\ge 2$ we give a class of geometric intersection graphs that contains all $(d-1)$-ball graphs and all unit *d*-ball graphs. We conjecture that for $d=3$, there exists an EPTAS for computing a maximum clique in the corresponding class. It is necessary that these superclasses be defined as classes of geometric intersection graphs. Indeed, it must be if we want to understand better the reason why efficient algorithms exist for both settings. For instance, taking the union of interval graphs and unit disk graphs would not give any insight, since it is a priori not defined by intersection graphs of some geometric objects.

**Fig. 1:**
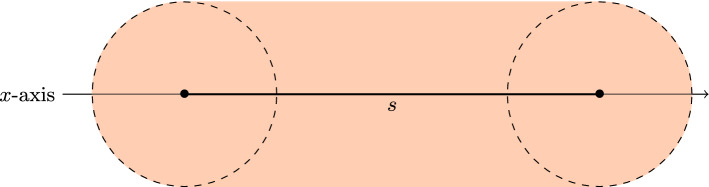
The union of the unit disks centred at points of *s* is a 2-pancake

In order to define the class, we first introduce the concept of *d*-*pancakes*. A 2-pancake is defined as the union of all unit disks whose centres lie on a line segment *s*, with *s* itself lying on the *x*-axis. An example is depicted in Fig. 1. This definition is equivalent to the Minkowski sum of a unit disk centred at the origin and a line segment on the *x*-axis, where the Minkowski sum of two sets *A*, *B* is defined as the set $\{a+b\mid a\in A, b \in B\}$. Similarly a 3-pancake is the union of all unit balls whose centres lie on a disk ${\mathcal {D}}$, with ${\mathcal {D}}$ lying on the *xy*-plane. More generally, we have:

### Definition 1

A *d*-*pancake* is a *d*-dimensional geometric object. Let us denote by $\{ \xi _1, \xi _2, \dots , \xi _d \}$ the canonical basis of ${\mathbb {R}}^d$. A *d*-pancake is defined as the Minkowski sum of the unit *d*-ball centred at the origin and a $(d-1)$-ball in the hyperspace induced by $\{ \xi _1, \xi _2, \dots , \xi _{d-1} \}$.

We denote by $\varPi ^d$ the class of intersection graphs of some finite collection of *d*-pancakes and unit *d*-balls. In this paper, we give a polynomial time algorithm for solving maximum clique in $\varPi ^2$: the intersection graphs class of unit disks and 2-pancakes. This is to put in contrast with the fact that computing a maximum clique in intersection graphs of unit disks and axis-parallel rectangles (instead of 2-pancakes) is NP-hard and even APX-hard, as shown together with Bonnet and Miltzow (2020), even though maximum clique can be solved in polynomial time in axis-parallel rectangle graphs (Imai and Asano 1983).

Relatedly, it would be interesting to generalise the existence of an EPTAS for maximum clique to superclasses of disk graphs. This was achieved with Bonnet and Miltzow for intersection graphs of homothets of a fixed bounded centrally symmetric convex set (Bonnet et al. 2020). In this paper, we aim at generalising further to intersection graphs of convex pseudo-disks, for which we conjecture the existence of an EPTAS, and give partial results towards proving it. The proof of these partial results relies on geometric permutations of line transversals. We do a case analysis on the existence of certain geometric permutations, and show that some convex pseudo-disks must intersect. Holmsen and Wenger have written a survey on geometric transversals (Wenger and Holmsen 2017). The results that are related to line transversals are either of Hadwiger-type, concerned with the conditions of existence of line transversals, or about the maximum number of geometric permutations of line transversals. To the best of our knowledge, we do not know of any result that uses geometric permutations of line transversals to show something else. We consider this tool, together with the polynomial time algorithm for computing a maximum clique in $\varPi ^2$, to be our main contributions.

## Preliminaries

### Graph notations

Let *G* be a simple graph. We say that two vertices are *adjacent* if there is an edge between them, otherwise they are *independent*. For a vertex *v*, the set ${\mathcal {N}}(v)$ denotes its *neighbourhood*, i.e. the set of vertices adjacent to *v*. We denote by $\omega (G)$, $\alpha (G)$, and $\chi (G)$ the clique number, the independence number and the chromatic number of *G*, respectively.

We denote by *V*(*G*) the vertex set of *G*. Let *H* be a subgraph of *G*. We denote by $G \setminus H$ the subgraph induced by $V(G) \setminus V(H)$. We denote by ${\overline{G}}$ the *complement* of *G*, which is the graph with the same vertex set, but where edges and non-edges are interchanged. A *bipartite graph* is graph whose vertex set can be partitioned into two independent sets. A graph is *cobipartite* if its complement is a bipartite graph.

We denote by $\text {iocp}(G)$ the *induced odd cycle packing number* of *G*, i.e. the maximum number of vertex-disjoint induced odd cycles (for each cycle the only edges are the ones making the cycle), such that there is no edge between two vertices of different cycles.

### Geometric notations

Throughout the paper we only consider Euclidean spaces with the Euclidean distance. Let *p* and $p'$ be two points in ${\mathbb {R}}^d$. We denote by $(p,p')$ the line going through them, and by $[p,p']$ the line segment with endpoints *p* and $p'$. We denote by $d(p,p')$ the distance between *p* and $p'$. For any fixed *d*, we denote by *O* the origin in ${\mathbb {R}}^d$. When $d=2$, we denote by *Ox* and *Oy* the *x* and *y*-axis, respectively. For $d=3$, we denote by *xOy* the *xy*-plane. We usually denote a *d*-pancake by $P^d$. As a reminder, a 2-pancake is the Minkowski sum of the unit disk centred at the origin *O* and a line segment lying on the axis *Ox*.

#### Definition 2

Let $\{S_i\}_{1\le i\le n}$ be a family of subsets of ${\mathbb {R}}^d$. We denote the *intersection graph* of $\{S_i\}$ by $G(\{ S_i\})$. It is the graph whose vertex set is $\{ S_i \mid 1 \le i \le n \}$ and where there is an edge between two vertices if and only if the corresponding sets intersect.

#### Definition 3

In ${\mathbb {R}}^2$ we denote by ${\mathcal {D}}(c,\rho )$ a closed disk centred at *c* with radius $\rho$. Let ${\mathcal {D}}={\mathcal {D}}(c,\rho )$ and ${\mathcal {D}}'={\mathcal {D}}(c',\rho ')$ be two intersecting disks. We call *lens induced by* ${\mathcal {D}}$ *and* ${\mathcal {D}}'$ the region ${\mathcal {D}}\cap {\mathcal {D}}'$. We call *half-lenses* the two closed regions obtained by dividing the lens along the line $(c,c')$.

For any $x_1\le x_2$, we denote by $P^2(x_1,x_2)$ the 2-pancake that is the Minkowski sum of the unit disk centred at *O* and the line segment with endpoints $x_1$ and $x_2$. Therefore we have $P^2(x_1,x_2) =\bigcup _{x_1 \le x' \le x_2} {\mathcal {D}}((x',0),1)$. Behind the definition of the *d*-pancakes is the idea that they should be as similar as possible to unit *d*-balls. In particular 2-pancakes should behave as much as possible like unit disks. This is perfectly illustrated when the intersection of a 2-pancake and a unit disk is a lens, as the intersection of two unit disks would be.

#### Definition 4

Let $\{P^2_j\}_{1 \le j \le n}$ be a set of 2-pancakes. For any unit disk ${\mathcal {D}}$, we denote by $L({\mathcal {D}},\{P^2_j\})$, or simply by $L({\mathcal {D}})$ when there is no risk of confusion, the set of 2-pancakes in $\{P^2_j\}$ whose intersection with ${\mathcal {D}}$ is a lens.

Let ${\mathcal {D}}$ denote ${\mathcal {D}}(c,1)$ for some point *c*. Observe that if a 2-pancake $P^2(x_1,x_2)$ for some $x_1 \le x_2$ is in $L({\mathcal {D}})$, then the intersection between ${\mathcal {D}}$ and $P^2(x_1,x_2)$ is equal to ${\mathcal {D}}\cap {\mathcal {D}}((x_1,0),1)$ or ${\mathcal {D}}\cap {\mathcal {D}}((x_2,0),1)$. We make an abuse of notation and denote by $d({\mathcal {D}},P^2(x_1,x_2))$ the smallest distance between *c* and a point in the line segment $[x_1,x_2]$. Observe that if the intersection between ${\mathcal {D}}$ and $P^2(x_1,x_2)$ is equal to ${\mathcal {D}}\cap {\mathcal {D}}((x_1,0),1)$, then $d({\mathcal {D}},P^2(x_1,x_2))=d(c,(x_1,0))$, and otherwise $d({\mathcal {D}},P^2(x_1,x_2))=d(c,(x_2,0))$. The following observation gives a characterisation of when the intersection between a unit disk and a 2-pancake is a lens.

#### Observation 1

Let ${\mathcal {D}}((c_x,c_y),1)$ be a unit disk intersecting with a 2-pancake $P^2(x_1,x_2)$. Then their intersection is a lens if and only if ($c_x \le x_1$ or $c_x \ge x_2)$ and the interior of ${\mathcal {D}}((c_x,c_y),1)$ does not contain any point in $\{(x_1, \pm 1),(x_2, \pm 1)\}$.

The observation follows immediately from the fact that the intersection is a lens if and only if ${\mathcal {D}}((c_x,c_y),1)$ does not contain a point in the open line segment between the points $(x_1,-1)$ and $(x_2,-1)$, nor in the open line segment between the points $(x_1,1)$ and $(x_2,1)$.

## Results

Unless explicitly mentioned, the following results were stated in the preliminary version of this paper (Grelier 2020), but their proofs were omitted due to space constraints.

We answer in Sect. 4 the 2-dimensional version of the question asked by Bonamy et al. (2018): We present a polynomial time algorithm for computing a maximum clique in a geometric superclass of interval graphs and unit disk graphs.

### Theorem 1

There exists a polynomial time algorithm for computing a maximum clique in $\varPi ^2$, even without a representation.

A part of the proof of Theorem 1 appeared in the preleminary version of this paper (Grelier 2020). Kang and Müller (2012) have shown that for any fixed $d\ge 2$, the recognition of unit *d*-ball graphs is NP-hard, and even $\exists {\mathbb {R}}$-hard. We conjecture that it is also hard to test whether a graph is in $\varPi ^d$ for any fixed $d\ge 3$, and prove it for $d=2$.

### Theorem 2

Testing whether a graph is in $\varPi ^2$ is NP-hard, and even $\exists {\mathbb {R}}$-hard.

The proof of Theorem 2 figures in Sect. 5. It immediately implies that given a graph *G* in $\varPi ^2$, finding a representation of *G* with 2-pancakes and unit disks is NP-hard. Therefore having a robust algorithm as defined in Raghavan and Spinrad (2001) is of interest. The algorithm of Theorem 1 takes any abstract graph as input, and outputs a maximum clique or a certificate that the graph is not in $\varPi ^2$.

Our polynomial time algorithm for maximum clique in $\varPi ^2$ gives some insight to why the complexity of maximum clique in disk graphs is still unknown. The class of interval graphs is arguably small: there is no induced cycle of length at least 4. Likewise, one can say that the class of unit disk graphs is small, as there is no star with at least 6 leaves. However, with disks one can realise arbitrarily large induced cycles and stars. One could have wondered whether when looking for a geometric class of graphs, wanting both arbitrarily large induced cycles and stars would force too much complexity. Our results with $\varPi ^2$ shows that actually, this is not where the difficulty lies. Indeed, one can realise with unit disks and 2-pancakes arbitrarily large induced cycles and stars. To solve maximum clique in disk graphs, or to show NP-hardness, it seems a good idea to investigate what are the disk graphs that are not in $\varPi ^2$.

Concerning $\varPi ^3$, we conjecture the following:

### Conjecture 1

There exists an integer *K* such that for any graph *G* in $\varPi ^3$, we have $\text {iocp}({\overline{G}})\le K$.

We show in Sect. 7 that this would be sufficient to obtain an EPTAS.

### Theorem 3

If Conjecture 1 holds, there exists a randomised EPTAS for computing a maximum clique in $\varPi ^3$, even without a representation.

By construction the class $\varPi ^d$ contains all $(d-1)$-ball graphs and all unit *d*-ball graphs. Indeed a $(d-1)$-ball graph can be realised by replacing in a representation each $(d-1)$-ball by a *d*-pancake. In addition to this property, we want fast algorithms for maximum clique in $\varPi ^d$. The definition of $\varPi ^d$ may seem unnecessarily complicated. The most surprising part of the definition is probably the fact that we use *d*-pancakes instead of simply using $(d-1)$-balls restricted to be in the same hyperspace of ${\mathbb {R}}^d$. However, we show in Sect. 4.3 that our arguments for proving fast algorithms would not hold with such a definition.

We give partial results toward showing the existence of an EPTAS for maximum clique in intersection graphs of *convex pseudo-disks*. We say that a graph is a *convex pseudo-disk graph* if it is the intersection graph of convex sets in the plane such that the boundaries of every pair intersect at most twice. We denote by ${\mathcal {G}}$ the class of intersection graphs of convex pseudo-disks. A structural property used to show the existence of an EPTAS for disk graphs is that for any disk graph *G*, $\text {iocp}({\overline{G}})\le 1$. The proof of Bonnet et al. (2018) relies heavily on the fact that disks have centres. However, convex pseudo-disks do not, therefore adapting the proof in this new setting does not seem easy. While we were not able to extend this structural result to the class ${\mathcal {G}}$, we show a weaker property: The complement of a triangle and an odd cycle is a forbidden induced subgraph in ${\mathcal {G}}$. We write “complement of a triangle” to make the connection with $\text {iocp}$ clear, but note that actually the complement of a triangle is an independent set of three vertices. Below we state this property more explicitly.

### Theorem 4

Let *G* be in ${\mathcal {G}}$. If there exists an independent set of size 3, denoted by *H*, in *G*, and if for any $u\in H$ and $v \in G \setminus H$, the edge $\{u,v\}$ is an edge of *G*, then $G \setminus H$ is cobipartite.

A part of the proof of Theorem 4 appeared in the preliminary version of this paper (Grelier 2020). We thank the anonymous reviewer who found a flaw in one of the lemmas, that we fix in this version. Note that a cobipartite graph is not the complement of an odd cycle. Given the three pairwise non-intersecting convex pseudo-disks in *H*, we give a geometric characterisation of the two independent sets in the complement of $G \setminus H$. We conjecture that Theorem 4 is true even when *H* is the complement of any odd cycle, which implies:

### Conjecture 2

For any convex pseudo-disk graph *G*, we have $\text {iocp}({\overline{G}})\le 1$.

If Conjecture 2 holds, it is straightforward to obtain an EPTAS for maximum clique in convex pseudo-disks graphs, by using the method of Bonamy et al. (2018).

## Computing a maximum clique in $\varPi ^2$ in polynomial time

In this section we prove Theorem 1. We first give a proof when a representation is given. The idea of the algorithm is similar to the one of Clark et al. (1990). We prove that if *u* and *v* are the most distant vertices in a maximum clique, then ${\mathcal {N}}(u) \cap {\mathcal {N}}(v)$ is cobipartite. In a second part, we give a robust algorithm, meaning that it does not require a representation, using tools introduced by Raghavan and Spinrad (2001).

### Computing a maximum clique with a representation

In their proof, Clark, Colbourn and Johnson use the following fact: if *c* and $c'$ are two points at distance $\rho$, then the diameter of the half-lenses induced by ${\mathcal {D}}(c,\rho )$ and ${\mathcal {D}}(c',\rho )$ is equal to $\rho$. We prove here a similar result.

#### Lemma 1

Let *c* and $c'$ be two points at distance $\rho$, and let be $\rho ' \ge \rho$. Then the diameter of the half-lenses induced by ${\mathcal {D}}(c,\rho )$ and ${\mathcal {D}}(c',\rho ')$ is at most $\rho '$.

#### Proof

First note that if $\rho '> 2\rho$ then the half-lenses are half-disks of ${\mathcal {D}}(c,\rho )$. The diameter of these half-disks is equal to $2\rho$, which is smaller than $\rho '$. Let us now assume that we have $\rho ' \le 2 \rho$. The boundary of the lens induced by ${\mathcal {D}}(c,\rho )$ and ${\mathcal {D}}(c',\rho ')$ consists of two arcs. The line $(c,c')$ intersects exactly once with each arc. One of these two intersections is $c'$, we denote by $c''$ the other. Let us consider the disk ${\mathcal {D}}(c'',\rho ')$. Note that it contains the disk ${\mathcal {D}}(c,\rho )$. Therefore the lens induced by ${\mathcal {D}}(c,\rho )$ and ${\mathcal {D}}(c',\rho ')$ is contained in the lens induced by ${\mathcal {D}}(c'',\rho ')$ and ${\mathcal {D}}(c',\rho ')$, whose half-lenses have diameter $\rho '$. The claim follows from the fact that the half-lenses of the first lens are contained in the ones of the second lens. $\square$

Before stating the next lemma, we introduce the following definition:

#### Definition 5

Let $\{S_i\}_{1\le i\le n}$ and $\{S'_j\}_{1\le j\le n'}$ be two families of sets in ${\mathbb {R}}^2$. We say that $\{S_i\}$ and $\{S'_j\}$ *fully intersect* if for all $1\le i \le n$ and $1\le j \le n'$ the intersection between $S_i$ and $S'_j$ is not empty.

#### Lemma 2

Let ${\mathcal {D}}:={\mathcal {D}}(c,1)$ be a unit disk and let $P^2:=P^2(x_1,x_2)$ be in $L({\mathcal {D}})$. Let $\{{\mathcal {D}}_i\}$ be a set of unit disks that fully intersect with $\{{\mathcal {D}},P^2\}$, such that for any ${\mathcal {D}}_i$ we have $d({\mathcal {D}},{\mathcal {D}}_i) \le d({\mathcal {D}},P^2)$. Moreover if $P^2$ is in $L({\mathcal {D}}_i)$ we require $d({\mathcal {D}}_i,P^2) \le d({\mathcal {D}},P^2)$. Also let $\{P^2_j\}$ be a set of 2-pancakes that fully intersect with $\{{\mathcal {D}},P^2\}$, such that for any $P^2_j$ in $\{P^2_j\} \cap L(D)$, we have $d({\mathcal {D}},P^2_j) \le d({\mathcal {D}},P^2)$. Then $G(\{{\mathcal {D}}_i\} \cup \{P^2_j\})$ is cobipartite.

#### Proof

**Fig. 2:**
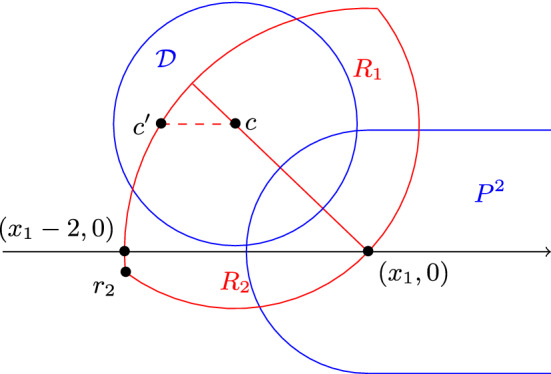
Illustration of the proof of Lemma 2

The proof is illustrated in Fig. 2. Without loss of generality, let us assume that the intersection between ${\mathcal {D}}$ and $P^2$ is equal to ${\mathcal {D}}\cap {\mathcal {D}}((x_1,0),1)$. Remember that by definition we have $x_1 \le x_2$. Let $P^2(x'_1,x'_2)$ be a 2-pancake in $\{P^2_j\}$. As it is intersecting with $P^2$, we have $x'_2\ge x_1-2$. Assume by contradiction that we have $x'_1 > x_1$. Then with Observation 1, we have that $P^2(x'_1,x'_2)$ is in $L({\mathcal {D}})$ and $d({\mathcal {D}},P^2(x'_1,x'_2))> d({\mathcal {D}},P^2)$, which is impossible. Therefore we have $x'_1 \le x_1$, and so $P^2(x'_1,x'_2)$ must contain ${\mathcal {D}}((x',0),1)$ for some $x'$ satisfying $x_1-2 \le x' \le x_1$. As the line segment $[(x_1-2,0),(x_1,0)]$ has length 2, the 2-pancakes in $\{P^2_j\}$ pairwise intersect.

We denote by $\rho$ the distance $d({\mathcal {D}},P^2)$. Let ${\mathcal {D}}(c_i,1)$ be a unit disk in $\{{\mathcal {D}}_i\}$. By assumption, $c_i$ is in ${\mathcal {D}}(c,\rho ) \cap {\mathcal {D}}((x_1,0),2)$. We then denote by *R* the lens that is induced by ${\mathcal {D}}(c,\rho )$ and ${\mathcal {D}}((x_1,0),2)$. We cut the lens into two parts with the line $(c,(x_1,0))$, and denote by $R_1$ the half-lens that is not below this line, and by $R_2$ the half-lens that is not above it. With Lemma 1, we obtain that the diameter of $R_1$ and $R_2$ is at most 2. Let us assume without loss of generality that *c* is not below *Ox*. We denote by $X_1$ the set of unit disks in $\{{\mathcal {D}}_i\}$ whose centre is in $R_1$. We denote by $X_2$ the union of $\{P^2_j\}$ and of the set of unit disks in $\{{\mathcal {D}}_i\}$ whose centre is in $R_2$. Since the diameter of $R_1$ is 2, any pair of unit disks in $X_1$ intersect, therefore $G(X_1)$ is a complete graph. To show that $G(X_2)$ is a complete graph too, it remains to show that any unit disk ${\mathcal {D}}(c_i,1)$ in $X_2$ and any 2-pancake $P^2(x'_1,x'_2)$ in $\{P^2_j\}$ intersect. We denote by $P^2_+$ the following convex shape: $\cup _{x'_1\le x \le x'_2}{\mathcal {D}}((x,0),2)$. Note that the fact that ${\mathcal {D}}(c_i,1)$ and $P^2(x'_1,x'_2)$ intersect is equivalent to having $c_i$ in $P^2_+$. Let us consider the horizontal line going through *c*, and let us denote by $c'$ the left intersection with the circle centred at $(x_1,0)$ with radius 2. We also denote by $r_2$ the extremity of *R* that is in $R_2$.

Let us assume by contradiction that $c_i$ is above the line segment $[c,c']$. As by assumption $c_i$ is in $R_2$, it implies that the *x*-coordinate of $c_i$ is smaller than the one of *c*. Therefore $P^2$ is in $L({\mathcal {D}}_i)$ and $d({\mathcal {D}}_i,P^2)>d({\mathcal {D}},P^2)$, which is impossible by assumption. Let us denote by $R_{2,-}$ the subset of $R_2$ that is not above the line segment $[c,c']$. To prove that ${\mathcal {D}}(c_i,1)$ and $P^2(x'_1,x'_2)$ intersect, it suffices to show that $P^2_+$ contains $R_{2,-}$. As shown above, $P^2(x'_1,x'_2)$ contains ${\mathcal {D}}((x',0),1)$ for some $x'$ satisfying $x_1-2 \le x' \le x_1$. This implies that $P^2_+$ contains ${\mathcal {D}}((x_1-2,0),2) \cap {\mathcal {D}}((x_1,0),2)$, and in particular contains $(x_1,0)$. Moreover as *c* is not below *Ox*, $r_2$ is also in ${\mathcal {D}}((x_1-2,0),2) \cap {\mathcal {D}}((x_1,0),2)$. As $P^2$ intersects ${\mathcal {D}}$, $P^2_+$ contains *c*. Let us assume by contradiction that $P^2_+$ does not contain $c'$. Then $x'_2$ must be smaller than the *x*-coordinate of $c'$, because otherwise the distance $d((x'_2,0),c')$ would be at most $d((x_1,0),c')$, which is equal to 2. But then if $P^2_+$ does not contain $c'$, then it does not contain *c* either, which is a contradiction. We have proved that $P^2_+$ contains the points $(x_1,0)$, *c*, $c'$ and $r_2$. By convexity, and using the fact that two circles intersect at most twice, we obtain that $R_{2,-}$ is contained in $P^2_+$. This shows that any two elements in $X_2$ intersect, which implies that $G(X_2)$ is a complete graph. Finally, as $X_1 \cup X_2 = \{{\mathcal {D}}_i\} \cup \{P^2_j\}$, we obtain that $G(\{{\mathcal {D}}_i\} \cup \{P^2_j\})$ can be partitioned into two cliques, i.e. it is cobipartite. $\square$

#### Lemma 3

Let ${\mathcal {D}}:={\mathcal {D}}((c_x,c_y),1)$ and ${\mathcal {D}}':={\mathcal {D}}((c'_x,c'_y),1)$ be two unit disks such that $c_x \le c_x'$. Let $P^2_1:=P^2(x_1,x_2)$ be a 2-pancake intersecting with ${\mathcal {D}}$ and ${\mathcal {D}}'$, such that $x_1 \ge c_x$ and $P^2_1$ is not in $L({\mathcal {D}})$. If $P^2_2:=P^2(x'_1,x'_2)$ is a 2-pancake intersecting with ${\mathcal {D}}$ and ${\mathcal {D}}'$, but not intersecting with $P^2_1$, then $P^2_2$ is in $L({\mathcal {D}}) \cap L({\mathcal {D}}')$.

#### Proof

**Fig. 3:**
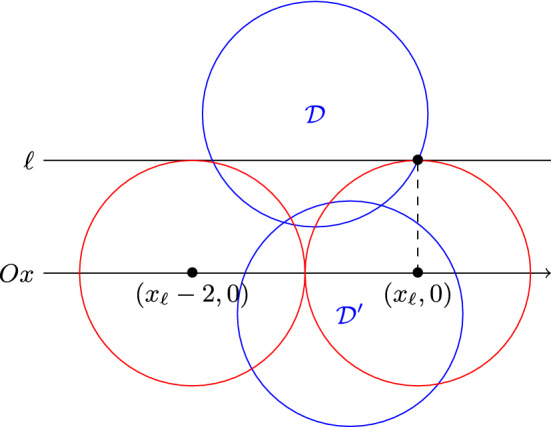
Illustration of the proof of Lemma 3

The proof is illustrated in Fig. 3. First let us prove that $P^2_2$ cannot be on the right side of $P^2_1$, i.e. we have $x'_1\le x_1$. Let us assume by contradiction $x'_1> x_1$. As $P^2_1$ and $P^2_2$ are not intersecting, we have $x'_1> x_1+2$. Hence, since we assume $x_1 \ge c_x$, we obtain $d(c,(x'_1,0))>2$, which is impossible. Therefore we have $x'_1\le x_1$, and even $x'_1< x_1-2$ since $P^2_1$ and $P^2_2$ are not intersecting.

Without loss of generality, let us assume $c_y\ge 0$. Let us consider the horizontal line $\ell$ with height 1. By assumption it intersects with the circle centred at $(c_x,c_y)$ with unit radius. There are at most two intersections, and we denote by $x_\ell$ the *x*-coordinate of the one to the right. As $P^2_1$ is not in $L({\mathcal {D}})$, we have $x_1\le x_\ell$. Then, since $x'_1< x_1-2$ and the fact that ${\mathcal {D}}$ has diameter 2, we know that the points $(x'_1,1)$ and $(x'_1,-1)$ are not in ${\mathcal {D}}$, which implies that $P^2_2$ is in $L({\mathcal {D}})$. Likewise as we have $c_x \le c_x'$, the points $(x'_1,1)$ and $(x'_1,-1)$ are not in ${\mathcal {D}}'$, and so $P^2_2$ is in $L({\mathcal {D}}) \cap L({\mathcal {D}}')$. $\square$

#### Lemma 4

Let ${\mathcal {D}}:={\mathcal {D}}(c,1)$ and ${\mathcal {D}}':={\mathcal {D}}(c',1)$ be two intersecting unit disks. Let $\{{\mathcal {D}}_i\}$ be a set of unit disks that fully intersect with $\{{\mathcal {D}},{\mathcal {D}}'\}$, such that for each unit disk ${\mathcal {D}}_i$ we have $d({\mathcal {D}},{\mathcal {D}}_i)\le d({\mathcal {D}},{\mathcal {D}}')$ and $d({\mathcal {D}}',{\mathcal {D}}_i)\le d({\mathcal {D}},{\mathcal {D}}')$. Also let $\{P^2_j\}$ be a set of 2-pancakes that fully intersect with $\{{\mathcal {D}},{\mathcal {D}}'\}$, such that for any $P^2_j$ in $\{P^2_j\} \cap L(D)$, we have $d({\mathcal {D}},P^2_j) \le d({\mathcal {D}},{\mathcal {D}}')$, and for any $P^2_j$ in $\{P^2_j\} \cap L(D')$, we have $d({\mathcal {D}}',P^2_j) \le d({\mathcal {D}},{\mathcal {D}}')$. Then $G(\{{\mathcal {D}}_i\} \cup \{P^2_j\})$ is cobipartite.

**Fig. 4:**
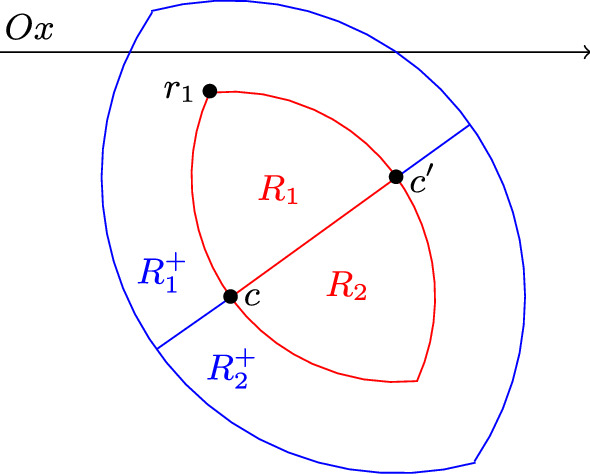
First case: $${\mathcal {D}}(c,2) \cap {\mathcal {D}}(c',2) \cap Ox \ne \emptyset $$

#### Proof

We denote by $\rho$ the distance between *c* and $c'$. We denote by *R* the lens induced by ${\mathcal {D}}(c,\rho )$ and ${\mathcal {D}}(c',\rho )$. We cut *R* with the line segment $[c,c']$, which partitions *R* into two half-lenses that we denote by $R_1$ and $R_2$. By assumption, the centre of any unit disk in $\{{\mathcal {D}}_i\}$ must be in *R*. Since $R_1$ and $R_2$ have diameter $\rho$ which is at most 2, any two unit disks having their centres in $R_1$ must intersect, and the same holds with $R_2$. Therefore $G(\{{\mathcal {D}}_i\})$ is cobipartite, which is the claim if $\{P^2_j\}$ is empty.

We now assume that $$\{P^2_j\}$$ is not empty. In order to show the claim, we do a case analysis according to whether the intersection between $${\mathcal {D}}(c,2) \cap {\mathcal {D}}(c',2)$$ and *Ox* is empty or not. Let us assume that the latter holds, as shown in Fig. 4. Let $$P^2$$ be in a 2-pancake in $$\{P^2_j\}$$. As $$P^2$$ intersects with $${\mathcal {D}}$$, $$P^2$$ contains a unit disk that intersects with $${\mathcal {D}}$$. Likewise, $$P^2$$ contains a unit disk that intersects with $${\mathcal {D}}'$$. This implies that $$P^2$$ contains a point in $${\mathcal {D}}(c,2) \cap Ox$$ and a point in $${\mathcal {D}}(c',2) \cap Ox$$. By convexity of a 2-pancake, $$P^2$$ contains a point $$(x',0)$$, where $$(x',0)$$ is in $${\mathcal {D}}(c,2) \cap {\mathcal {D}}(c',2)\cap Ox$$. We denote by $$R^+$$ the lens that is induced by $${\mathcal {D}}(c,2)$$ and $${\mathcal {D}}(c',2)$$ and cut it with the line $$(c,c')$$. We denote by $$R^+_1$$ (respectively $$R^+_2)$$ the half-lens that contains $$R_1$$ (respectively $$R_2$$). Let us assume that $$(x',0)$$ is in $$R^+_1$$. By assumption $${\mathcal {D}}((x',0),2)$$ contains *c* and $$c'$$. Let us consider the third extremity of $$R_1$$, along with *c* and $$c'$$, that we denote by $$r_1$$. By making a circle centred at $$r_1$$ grow, we observe that the farthest point from $$r_1$$ in $$R^+_1$$ can only be at one of the three extremities of $$R^+_1$$. However by Lemma 1 these distances are at most 2, which implies that the distance between $$(x',0)$$ and $$r_1$$ is at most 2. Using the fact that two circles intersect at most twice, we obtain that $$R_1$$ is contained in $${\mathcal {D}}((x',0),2)$$. Therefore $$P^2$$ intersect with all unit disks whose centre is in $$R_1$$, and with all 2-pancakes in $$L({\mathcal {D}}) \cap L({\mathcal {D}}')$$ that contain a disk whose centre is in $$R_1$$. Let $$P^2(x_1,x_2)$$ and $$P^2(x'_1,x'_2)$$ be two 2-pancakes in $$\{P^2_j\}$$ such that they contain each a unit disk whose centre is in $$R^+_1$$, but such that they do not contain a unit disk whose centre is in $$R_1$$. In particular, $$P^2(x_1,x_2)$$ and $$P^2(x'_1,x'_2)$$ are not in $$L({\mathcal {D}}) \cap L({\mathcal {D}}')$$. We claim that they intersect. Suppose by contradiction that they do not. Without loss of generality, let us assume that $$P^2(x_1,x_2)$$ is to the right of $$P^2(x'_1,x'_2)$$, and that $$c_x \le c'_x$$, where $$c_x$$ and $$c'_x$$ denote the *x*-coordinate of *c* and $$c'$$ respectively. Since $$P^2(x_1,x_2)$$ does not contain a disk in $$R_1$$, and since it is on the right side of $$P^2(x'_1,x'_2)$$, it implies that it does not contain a disk with centre in $${\mathcal {D}}(c,\rho )$$. Therefore $$P^2(x_1,x_2)$$ cannot be in $$L({\mathcal {D}})$$. Moreover the fact that it does not contain a disk with centre in $${\mathcal {D}}(c,\rho )$$ implies $$x_1\ge c_x$$. We finally apply Lemma 3 to obtain a contradiction. We denote $$X_1$$ the set of unit disks whose centre is in $$R_1$$ and 2-pancakes that contain a disk whose centre is in $$R^+_1$$. We know that two unit disks in $$X_1$$ intersect. Moreover we have shown that a 2-pancake and a unit disk in $$X_1$$ intersect. For a pair of two pancakes, if one of them contains a disk whose centre is in $$R_1$$ it is done for the same reasons. If none of them does, then we have shown above that they intersect. This shows that $$G(X_1)$$ is a complete graph. By defining $$X_2$$ as the set of the remaining disks and 2-pancakes, using the symmetry of the problem we obtain that $$G(X_2)$$ is also a complete graph.

**Fig. 5:**
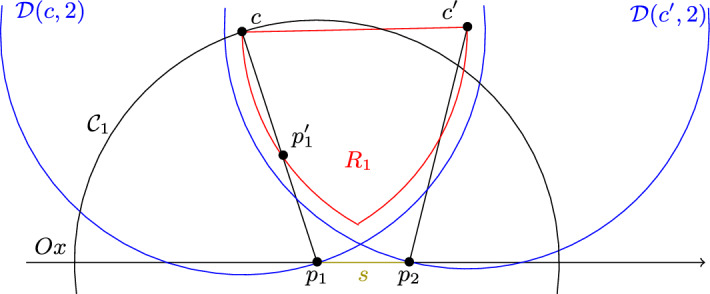
Second case: $${\mathcal {D}}(c,2) \cap {\mathcal {D}}(c',2) \cap Ox= \emptyset $$

Now let us assume that the intersection between ${\mathcal {D}}(c,2) \cap {\mathcal {D}}(c',2)$ and *Ox* is empty, as shown in Fig. 5. As $\{P^2_j\}$ is not empty, the set $Ox \setminus ({\mathcal {D}}(c,2) \cup {\mathcal {D}}(c',2))$ consists of three connected component, one of them bounded. We denote by *s* the closed line segment consisting of the bounded connected component and its boundaries. Any 2-pancake $P^2$ in $\{P^2_j\}$ contains a unit disk whose centre is in ${\mathcal {D}}(c,2) \cap Ox$, otherwise $P^2$ would not intersect with ${\mathcal {D}}$. Likewise $P^2$ contains a unit disk whose centre is in ${\mathcal {D}}(c',2) \cap Ox$, and therefore $P^2$ contains *s*. This implies that all 2-pancakes in $\{P^2_j\}$ pairwise intersect. Let us assume without loss of generality that $R_1$ is closer to *Ox* than $R_2$. Let us show that any 2-pancake $P^2$ in $\{P^2_j\}$ and any unit disk whose centre is in $R_1$ intersect. This is equivalent to show that for any point *p* in $R_1$, there exists a unit disk contained in $P^2$ with centre $q \in P^2 \cap Ox$, such that the Euclidean distance between *p* and *q* is at most 2. We denote by $P^2_+$ the Minkowski sum of the disk with radius 2 centred at *O* and the line segment *s*, i.e. $P^2_+=\cup _{x' \in s}{\mathcal {D}}(x',2)$. Note that $P^2_+$ is convex. We claim that $P^2_+$ contains $R_1$, which implies the desired property. Since *s* contains a point $p_1$ in ${\mathcal {D}}(c,2)$, we know that $P^2_+$ contains *c*. Likewise, as *s* contains a point $p_2$ in ${\mathcal {D}}(c',2)$, then $P^2_+$ contains $c'$, and therefore by convexity the whole line segment $[c,c']$. Therefore $P^2_+$ contains the quadrilateral $cc'p_2p_1$. If this quadrilateral contains $R_1$ we are done. Otherwise, it may not contain a circular segment of the disk ${\mathcal {D}}(c',\rho )$ or a circular segment of the disk ${\mathcal {D}}(c,\rho )$. Let us assume that we have the worst case, meaning that both circular segments are not in $cc'p_2p_1$. Let us consider the circle ${\mathcal {C}}_1$ centred at $p_1$ with radius 2, and the circle ${\mathcal {C}}'$ centred at $c'$ with radius $\rho$. The two circles intersect at *c*. Let us consider the point $p'_1$ that is at the intersection between ${\mathcal {C}}'$ and the line segment $[c,p_1]$. By definition, $p'_1$ is inside the disk induced by ${\mathcal {C}}_1$. As two circles intersect at most twice, we obtain that the arc $cp'_1$ centred at $c'$ with radius $\rho$ is contained in the disk induced by ${\mathcal {C}}_1$, and therefore also in $P^2_+$. By convexity, we know that the circular segment of the disk ${\mathcal {D}}(c',\rho )$ with chord $[c,p'_1]$ is in $P^2_+$. We can apply the same arguments for the other side to show that $R_1$ is in $P^2_+$. Hence by defining $X_1$ as the set of disks whose centre centre is in $R_1$, union the set of 2-pancakes, and $X_2$ as the set of disks whose centre is in $R_2$, we have that $G(X_1)$ and $G(X_2)$ are complete graphs. $\square$

Note that Lemmas 2 and 4 give a polynomial time algorithm for maximum clique in $\varPi ^2$ when a representation is given. First compute a maximum clique that contains only 2-pancakes, which can be done in polynomial time since the intersection graph of a set of 2-pancakes is an interval graph (Gupta et al. 1982). Then for each unit disk ${\mathcal {D}}$, compute a maximum clique which contains exactly one unit disk, ${\mathcal {D}}$, and an arbitrary number of 2-pancakes. Because finding out whether a unit disk and a 2-pancake intersect takes constant time, computing such a maximum clique can be done in polynomial time. Note that if a maximum clique contains at least two unit disks, then in quadratic time we can find in this maximum clique either a pair of unit disks or a unit disk and a 2-pancake whose intersection is a lens, such that the conditions of Lemmas 2 or of 4 are satisfied. By applying the corresponding lemma, we know that we are computing a maximum clique in a cobipartite graph, which is the same as computing a maximum independent set in a bipartite graph. As this can be done in polynomial time (Edmonds and Karp 1972), we can compute a maximum clique in $\varPi ^2$ in polynomial time when the representation is given.

### Computing a maximum clique without a representation

To obtain an algorithm that does not require a representation, we use the notion of cobipartite neighbourhood edge elimination ordering (CNEEO) as introduced by Raghavan and Spinrad (2001). Let *G* be a graph with *m* edges. Let $\varLambda =e_1,e_2, \dots , e_m$ be an ordering of the edges. Let $G_\varLambda (k)$ be the subgraph of *G* with edge set $\{e_k,e_{k+1}, \dots , e_m\}$. For each $e_k=(u,v)$, $N_{\varLambda ,k}$ is defined as the set of vertices adjacent to *u* and *v* in $G_\varLambda (k)$.

#### Definition 6

(Raghavan and Spinrad 2001) We say that an edge ordering $\varLambda =\{e_1,e_2,\dots ,e_m\}$ is a CNEEO if for each $e_k$, $N_{\varLambda ,k}$ induces a cobipartite graph in *G*.

#### Lemma 5

(Raghavan and Spinrad 2001) Given a graph *G* and a CNEEO $\varLambda$ for *G*, a maximum clique in *G* can be found in polynomial time.

They propose a greedy algorithm for finding a CNEEO: When having chosen the first $i-1$ edges $e_1, \ldots , e_{i-1}$, try every remaining edge one by one until finding one that satisfies the required property. If no such edge exists, return that the graph does not admit a CNEEO, which follows from Lemma 6.

#### Lemma 6

(Raghavan and Spinrad 2001) If *G* admits a CNEEO, then the greedy algorithm finds a CNEEO for *G*.

To show that it is possible to compute a maximum clique in a graph *G* in $\varPi ^2$, we show that such a graph admits a CNEEO. As noted by Raghavan and Spinrad, the algorithm computes a maximum clique for any graph that admits a CNEEO, and otherwise states that the given graph does not admit a CNEEO. In particular, the algorithm does not say whether the graph is indeed in $\varPi ^2$, and cannot be used for recognition.

#### Theorem 5

If a graph *G* is in $\varPi ^2$, then *G* admits a CNEEO.

Theorem 5, Lemmas 5 and 6 immediately imply Theorem 1. To prove Theorem 5, we use two more lemmas.

#### Lemma 7

Let ${\mathcal {D}}={\mathcal {D}}((c_x,c_y),1)$ be a unit disk. Let $\{P^2_j\}$ be a set of 2-pancakes that all intersect with ${\mathcal {D}}$. Then $G(\{P^2_j\})$ is cobipartite.

#### Proof

Let $P^2(x_1,x_2)$ be in $\{P^2_j\}$. By triangular inequality we have $x_1 \le c_x+2$ or $x_2 \ge c_x-2$. It implies that $P^2(x_1,x_2)$ contains the line segment $[(x'-1,0),(x'+1,0)]$ for some $x'$ satisfying $c_x-2 \le x' \le c_x+2$. We define $X_1$ as the set of 2-pancakes in $\{P^2_j\}$ that contain the line segment $[(x'-1,0),(x'+1,0)]$ for some $x'$ satisfying $c_x-2 \le x' \le c_x$, and $X_2$ as $\{P^2_j\} \setminus X_1$. We obtain that $G(X_1)$ and $G(X_2)$ are complete graphs. $\square$

#### Lemma 8

Let $P^2=P^2(x_1,x_2)$ and $P'^2=P^2(x'_1,x'_2)$ be two intersecting 2-pancakes. Let $\{P^2_j\}$ be a set of 2-pancakes that fully intersect with $\{P^2,P'^2\}$, such that for any $P^2_j$ in $\{P^2_j\}$, $P^2_j$ is not contained in $P^2$ nor in $P'^2$. Then $G(\{P^2_j\})$ is cobipartite.

#### Proof

Let $P^2(x''_1,x''_2)$ be in $\{P^2_j\}$. Let us first assume that one of $P^2,P'^2$ is contained in the other. Without loss of generality, let us assume that $P^2$ is contained in $P'^2$, which is equivalent to having $x'_1\le x_1 \le x_2 \le x'_2$. By assumption, as $P^2(x''_1,x''_2)$ is not contained in $P^2$, we have $x''_1<x_1$ or $x_2<x''_2$. As $P^2(x''_1,x''_2)$ intersects with $P^2$, it implies that $P^2(x''_1,x''_2)$ contains $(x_1-1,0)$ or $(x_2+1,0)$. We define $X_1$ as the set of 2-pancakes in $\{P^2_j\}$ that contains $(x_1-1,0)$, and $X_2$ as $\{P^2_j\}\setminus X_1$. We obtain that $G(X_1)$ and $G(X_2)$ are complete graphs.

If none of $P^2,P'^2$ is contained in the other, we can assume without loss of generality that $x_1 \le x'_1 \le x_2 \le x'_2$. Therefore we have $x''_1<x'_1$ or $x_2<x''_2$, which implies that $P^2(x''_1,x''_2)$ contains $(x'_1-1,0)$ or $(x_2+1,0)$. We conclude as above. $\square$

#### Proof of Theorem 5

Let us consider any representation of *G* with unit disks and 2-pancakes. We divide the set of edges into three sets: $E_1$, $E_2$ and $E_3$. $E_1$ contains all the edges between a pair of unit disks, or between a unit disk ${\mathcal {D}}$ and a 2-pancake in *L*(*D*). $E_2$ contains the edges between a unit disk and a 2-pancake that are not in $E_1$. $E_3$ contains the edges between a pair of 2-pancakes. For an edge $e=\{u,v\}$ in $E_1$, we call length of *e* the distance between *u* and *v*, be they unit disks or a unit disk ${\mathcal {D}}$ and a 2-pancake in *L*(*D*). We order the edges in $E_1$ by non increasing length, which gives an ordering $\varLambda _1$. We take any ordering $\varLambda _2$ of the edges in $E_2$. For $E_3$, we take any ordering $\varLambda _3$ such that for any edge $e=\{u,v\}$, no edge after *e* in $\varLambda _3$ contains a 2-pancake contained in *u* or *v*. This can be obtained by considering the smallest 2-pancakes first. We finally define an ordering $\varLambda = \varLambda _1 \varLambda _2 \varLambda _3$ on *E*. Let us consider an edge $e_k$. If $e_k$ is in $E_1$, Lemmas 2 and 4 show that $N_{\varLambda ,k}$ induces a cobipartite graph. If $e_k$ is in $E_2$, we use Lemma 7, and if $e_k$ is in $E_3$, we conclude with Lemma 8. This shows that $\varLambda$ is a CNEEO. $\square$

### A motivation for $\varPi ^d$

As we define it, $\varPi ^d$ is the class of intersection graphs of *d*-pancakes and unit *d*-balls. The properties that we desire are:

1. $\varPi ^d$ contains $(d-1)$-ball graphs and unit *d*-ball graphs,
2. Maximum clique can be computed as fast in $\varPi ^d$ as in $(d-1)$-ball graphs and unit *d*-ball graphs.

Let $\{\xi _i\}_{1 \le i \le d}$ be the canonical basis of ${\mathbb {R}}^d$. Let us consider another class ${\tilde{\varPi }}^d$, that might a priori satisfy those properties.

#### Definition 7

We denote by ${\tilde{\varPi }}^d$ the class of intersection graphs of $(d-1)$-balls lying on the hyperspace induced by $\{ \xi _1, \xi _2, \dots , \xi _{d-1} \}$ and of unit *d*-balls.

This class might look more natural since it makes use only of balls and not of pancakes. It contains by definition $(d-1)$-ball graphs and unit *d*-ball graphs. Moreover, as we want to be able to compute a maximum clique fast, we are looking for a “small” superclass. However, while we do not rule out the existence of a polynomial algorithm for computing a maximum clique in ${\tilde{\varPi }}^2$, we demonstrate that Lemma 4 does not hold in ${\tilde{\varPi }}^2$.

**Fig. 6:**
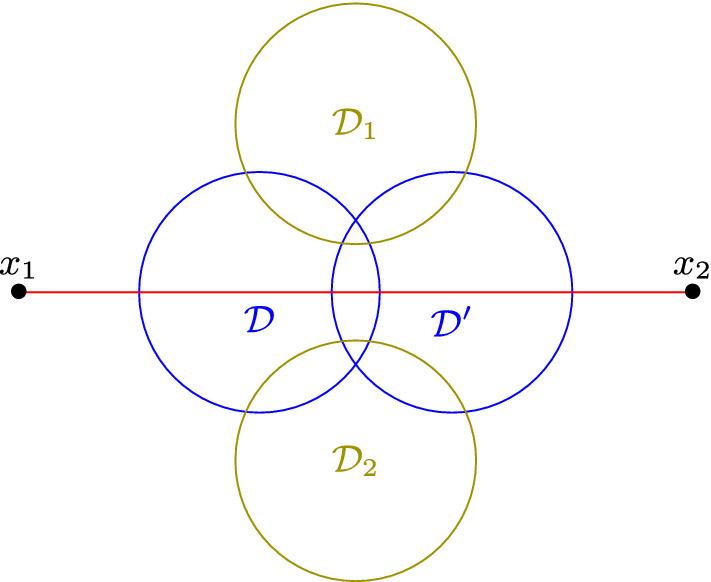
Lemma 4 does not hold in ${\tilde{\varPi }}^2$: $G(\{{\mathcal {D}}_1,{\mathcal {D}}_2,[x_1,x_2]\})$ is not cobipartite

The counterexample is illustrated in Fig. 6. We have two intersecting unit disks ${\mathcal {D}}$ and ${\mathcal {D}}'$. Moreover each one of ${\mathcal {D}}_1$, ${\mathcal {D}}_2$ and the line segment $[x_1,x_2]$ intersects with both ${\mathcal {D}}$ and ${\mathcal {D}}'$. The distances $d({\mathcal {D}},{\mathcal {D}}_1)$, $d({\mathcal {D}}',{\mathcal {D}}_1)$ are smaller than $d({\mathcal {D}},{\mathcal {D}}')$, and the same hold for ${\mathcal {D}}_2$. To define $L({\mathcal {D}})$, a natural way would be to use the same characterisation as in Observation 1. Therefore the line segment $[x_1,x_2]$ is not in $L({\mathcal {D}})$ nor in $L({\mathcal {D}}')$. However, $G(\{{\mathcal {D}}_1,{\mathcal {D}}_2,[x_1,x_2]\})$ is an edgeless graph with three vertices, and therefore is not cobipartite.

## Recognition of graphs in $\varPi ^2$

We show that testing whether a graph can be obtained as the intersection graph of unit disks and 2-pancakes is hard, as claimed in Theorem 2.

### Proof of Theorem 2

We do a reduction from recognition of unit disk graphs, which is $\exists {\mathbb {R}}$-hard as shown by Kang and Müller (2012). Let $G=(V,E)$ be a graph with *n* vertices. We are going to construct $$\left( {\begin{array}{c}n\\ 2\end{array}}\right) $$ graphs such that *G* is a unit disk graph if and only if at least one of these new graphs is in $\varPi ^2$. Let *S* and $S'$ be two stars with internal vertex *s* and $s'$ respectively, having $14n+8$ leaves each. Let *W* and $W'$ be two paths with 2*n* vertices each with end vertices $w_1,w_{2n}$ and $w'_1,w'_{2n}$ respectively. Let *u* and *v* be two vertices in *V*. We define $G_{u,v}$ as the graph obtained by connecting *s* to $s'$, $w_1$ to *u*, $w'_1$ to *v*, $w_{2n}$ to *s* and $w'_{2n}$ to $s'$. We claim that *G* is a unit disk graph if and only if $G_{u,v}$ is in $\varPi ^2$ for some *u* and *v* in *V*. First let us assume that *G* is a unit disk graph. Let us consider the set *P* of the centres of the unit disks in any fixed representation of *G*. Consider two extreme points in *P*, meaning that removing any of them modifies the convex hull of the point set. Take the two unit disks ${\mathcal {D}}$ and ${\mathcal {D}}'$ corresponding to those two extreme points, and let us denote by *u* and *v* the corresponding vertices in *G*. Now take two sets $\{{\mathcal {D}}_i\}_{1 \le i \le 2n}$ and $\{{\mathcal {D}}'_j\}_{1 \le j \le 2n}$ of 2*n* unit disks such that $G(\{{\mathcal {D}}_i\})$ and $G(\{{\mathcal {D}}'_j\})$ are paths, and such that no two unit disks of the form ${\mathcal {D}}_i,{\mathcal {D}}'_j$ intersect. Moreover we require that $G(\{{\mathcal {D}}_i\})\cap G = (\{u\}, \emptyset )$ and $G(\{{\mathcal {D}}_j\})\cap G = (\{v\},\emptyset )$, and that all unit disks centres are on the same side of the line $(c_{2n},c'_{2n})$, which are the centres of ${\mathcal {D}}_{2n}$ and ${\mathcal {D}}'_{2n}$ respectively. This is possible because the most distant points in the unit disk representation of *G* have distance at most 4*n*, and we have 2*n* unit disks in each path. Then we translate and rotate everything so that the *y*-coordinate of $c_{2n}$ and $c'_{2n}$ is equal to 2, and that all other centres are above the horizontal line with height 2. We take two intersecting 2-pancakes such that one also intersect with ${\mathcal {D}}_{2n}$ and the other with ${\mathcal {D}}'_{2n}$. We choose these 2-pancakes big enough so that for each of them we can add $14n+8$ pairwise non intersecting unit disks, but intersecting with their respective 2-pancake. This shows that if *G* is a unit disk graph, then $G_{u,v}$ is in $\varPi ^2$.

Let us now assume that $G_{u,v}$ is in $\varPi ^2$, for some *u*, *v* in *V*. As a unit disk can intersect at most with 5 pairwise non intersecting unit disks, we have that in any $\varPi ^2$ representation of $G_{u,v}$, *s* and $s'$ must be represented by 2-pancakes, denoted by *P* and $P'$ respectively. Let *x* be the length of the line segment obtained as the intersection of *P* and *Ox*. Note that all points of a unit disk intersecting a 2-pancake are within distance 3 of *Ox*. Therefore, the unit disks corresponding to leaves of *s* are contained in a rectangle with area $6(x+4)$. Moreover, for each 2-pancake intersecting *P*, there is a unit disk contained in this 2-pancake that intersects *P*. We have $14n+8$ pairwise non-intersecting unit disks in a rectangle with area $6(x+4)$. As the area of a unit disk is bigger than 3, we have $6(x+4)\ge 3(14n+8)$, or equivalently $x\ge 7n$. Note that the same holds with $P'$. Let us show that in any $\varPi ^2$ representation of $G_{u,v}$, all the vertices in *V* are represented by unit disks. Assume by contradiction that it is not the case. Without loss of generality, let us assume *P* is to the left of $P'$, and that one vertex $u_G$ in *V* is represented by a 2-pancake that is to the right of $P'$. Indeed this 2-pancake cannot be between *P* and $P'$ because they are intersecting. Let us consider the last vertex in a path from *s* to $u_G$ that is a disk. By construction, the distance between *P* and the unit disk corresponding to this vertex is at most $2(2n+n-1)=6n-2$. This shows that this vertex is still far from the right end of $P'$, and so the next vertex has to be represented by a unit disk because it is not intersecting $P'$, which is a contradiction. We have shown that *G* is a unit disk graph if and only if there exist *u*, *v* in *V* such that $G_{u,v}$ is in $\varPi ^2$, and the construction of these $$\left( {\begin{array}{c}n\\ 2\end{array}}\right) $$ graphs takes linear time for each of them. $\square$

## Intersection graphs of convex pseudo-disks

In this section we are interested in computing a maximum clique in intersection graphs of convex pseudo-disks. As mentioned in the introduction, there exists an EPTAS for maximum clique in disk graphs (Bonnet et al. 2018; Bonamy et al. 2018). The main property used is that for any disk graph *G*, we have $\text {iocp}({\overline{G}})\le 1$. The proof of this inequality relies on the fact that disks have centres. However in this section we are considering convex pseudo-disks, which do not have centres. Our proof that the complement of a triangle and an odd cycle is a forbidden induced subgraph in convex pseudo-disk graphs relies on line transversals and their geometric permutations on the three convex pseudo-disks that form a triangle in the complement, denoted by ${\mathcal {D}}_1$, ${\mathcal {D}}_2$ and ${\mathcal {D}}_3$. As there are only three sets, the geometric permutation of a line transversal is given simply by stating which set is the second one intersected. We denote by $\{ {\mathcal {D}}'_j\}_{1\le j\le n}$ (or simply $\{ {\mathcal {D}}'_j\}$) a set of convex pseudo-disks that fully intersect with $\{{\mathcal {D}}_1,{\mathcal {D}}_2,{\mathcal {D}}_3\}$. Our aim is to show that $G(\{ {\mathcal {D}}'_j\})$ is cobipartite.

Throughout this section, for the sake of readability, we refer to the convex pseudo-disks simply as “disks”. We always assume that no disk ${\mathcal {D}}\in \{ {\mathcal {D}}'_j\}$ contains any of ${\mathcal {D}}_1$, ${\mathcal {D}}_2$ and ${\mathcal {D}}_3$. Indeed, ${\mathcal {D}}$ would intersect any disk that intersects pairwise with ${\mathcal {D}}_1$, ${\mathcal {D}}_2$ and ${\mathcal {D}}_3$. Thus, such a disk ${\mathcal {D}}$ could be arbitrarily added to any of the two cliques of the cobipartition.

### Definition 8

A *line transversal* $\ell$ is a line that intersects each of the three disks ${\mathcal {D}}_1$, ${\mathcal {D}}_2$ and ${\mathcal {D}}_3$. We call *disk in the middle* of a line transversal the disk it intersects in second position.

We are going to conduct a case analysis depending on the number of disks being the disk in the middle for some line transversal. If there exists no line transversal, we can prove a stronger statement.

### Lemma 9

If there is no line transversal through a family of convex sets *F*, then for any pair of convex sets $\{C_1,C_2\}$ that fully intersects with *F*, the sets $C_1$ and $C_2$ intersect.

### Proof

Let us prove the contrapositive. Assume that $C_1$ and $C_2$ do not intersect, therefore there exists a separating line. As all sets in *F* intersect $C_1$ and $C_2$, they also intersect the separating line, which is thus a line transversal of *F*. $\square$

Using the notation of Theorem 4, Lemma 9 immediately implies that if there is no line transversal through the sets representing *H*, then $G\setminus H$ is a clique, which is an even stronger statement than required.

### Definition 9

Let ${\mathcal {D}}_1$ and ${\mathcal {D}}_2$ be two disjoint disks and let *p*, *q* be in the interior of ${\mathcal {D}}_1, {\mathcal {D}}_2$ respectively. We call *external tangents* of ${\mathcal {D}}_1$ and ${\mathcal {D}}_2$ the two tangents that do not cross the line segment [*p*, *q*].

**Fig. 7:**
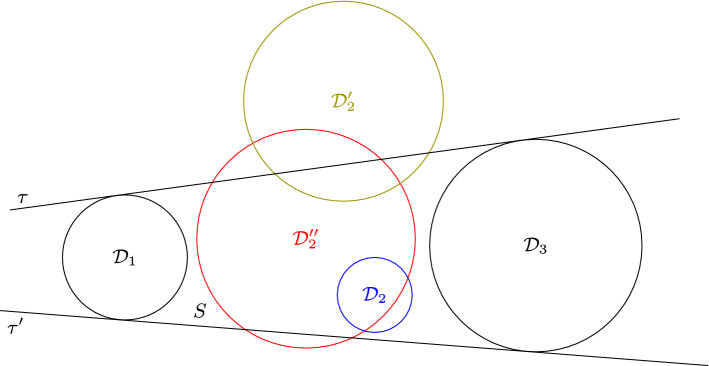
${\mathcal {D}}_2$ is contained, ${\mathcal {D}}'_2$ is 1-intersecting and ${\mathcal {D}}''_2$ is 2-intersecting

### Definition 10

Let us consider a disk in $\{{\mathcal {D}}_1,{\mathcal {D}}_2,{\mathcal {D}}_3\}$, say ${\mathcal {D}}_2$, such that it is the disk in the middle of a line transversal. We denote by $\tau$ and $\tau '$ the two external tangents of ${\mathcal {D}}_1$ and ${\mathcal {D}}_3$. We say that ${\mathcal {D}}_2$ is *contained* if it is contained in the bounded region *S* delimited by ${\mathcal {D}}_1$, $\tau$, ${\mathcal {D}}_3$ and $\tau '$. If ${\mathcal {D}}_2$ intersects exactly one of the external tangents, we say that ${\mathcal {D}}_2$ is 1-*intersecting*. If ${\mathcal {D}}_2$ intersects both external tangents, we say that ${\mathcal {D}}_2$ is 2-*intersecting*. The different cases are illustrated in Fig. 7.

### Lemma 10

If ${\mathcal {D}}_2$ is 2-intersecting, then it is the disk in the middle of all line transversals.

### Proof

By definition, ${\mathcal {D}}_2$ is the disk in the middle of a line transversal. We denote by $\tau$ and $\tau '$ the external tangents. let *p* be a point in ${\mathcal {D}}_2 \cap \tau$ and $p'$ be in ${\mathcal {D}}_2 \cap \tau '$. The line segment $[p,p']$ is contained in ${\mathcal {D}}_2$, and the line $(p,p')$ separates ${\mathcal {D}}_1$ from ${\mathcal {D}}_3$. Let $\ell$ be a line transversal. Let $p_1$ be in $\ell \cap {\mathcal {D}}_1$ and $p_3$ be in $\ell \cap {\mathcal {D}}_3$. The line segment $[p_1,p_3]$ must cross $[p,p']$, which shows that the disk in the middle of $\ell$ is ${\mathcal {D}}_2$. $\square$

### Lemma 11

If ${\mathcal {D}}_2$ is contained, then either ${\mathcal {D}}_1$ is not the disk in the middle of a line transversal, or ${\mathcal {D}}_1$ is 1-intersecting. The same holds with ${\mathcal {D}}_3$.

### Proof

We prove the statement for ${\mathcal {D}}_1$, the same arguments hold with ${\mathcal {D}}_3$. Observe that there is a line transversal having ${\mathcal {D}}_2$ as disk in the middle. Let us assume that there is a line transversal having ${\mathcal {D}}_1$ as disk in the middle, and let us show that ${\mathcal {D}}_1$ is 1-intersecting. As ${\mathcal {D}}_2$ is contained, no point in ${\mathcal {D}}_2$ lies on the boundary of the convex hull of ${\mathcal {D}}_1 \cup {\mathcal {D}}_2 \cup {\mathcal {D}}_3$. This immediately implies that some points in ${\mathcal {D}}_1$ lies on the boundary of the convex hull of ${\mathcal {D}}_1 \cup {\mathcal {D}}_2 \cup {\mathcal {D}}_3$. Therefore, ${\mathcal {D}}_1$ is not contained. Moreover, ${\mathcal {D}}_1$ is not 2-intersecting, for otherwise ${\mathcal {D}}_2$ would not be the disk in the middle of a line transversal, as stated in Lemma 10. We have shown that ${\mathcal {D}}_1$ is 1-intersecting. $\square$

**Fig. 8:**
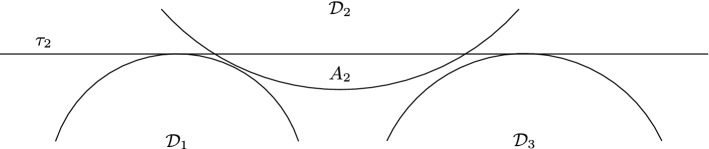
Illustration of Definition 11, with ${\mathcal {D}}_2$ being the 1-intersecting disk

**Fig. 9:**
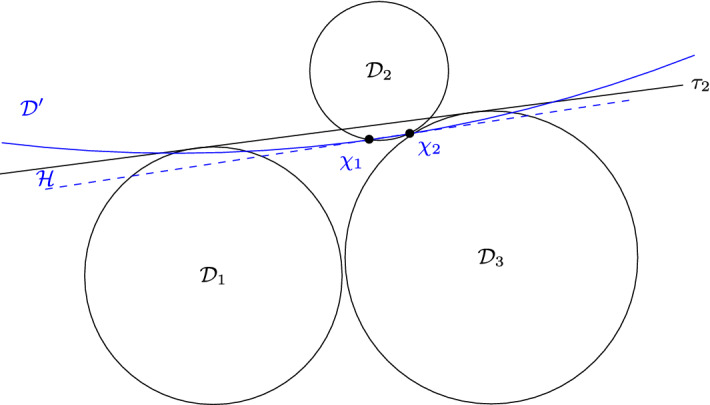
Illustration of Definition 11, ${\mathcal {D}}'$ is centred with respect to ${\mathcal {D}}_2$

**Fig. 10:**
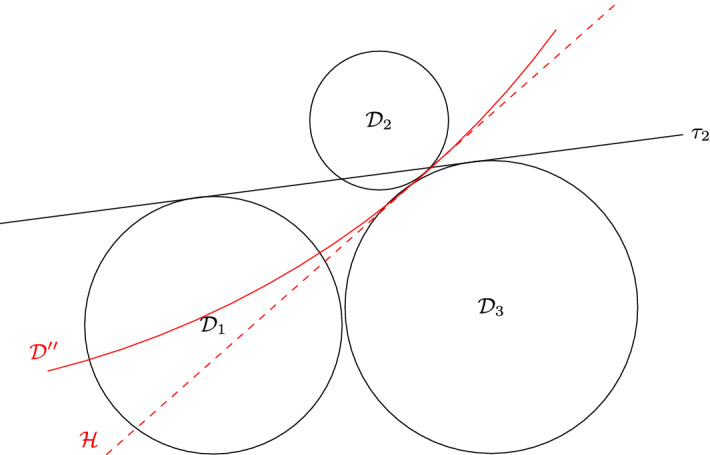
Illustration of Definition 11, ${\mathcal {D}}''$ is outside-containing ${\mathcal {D}}_2$, but not centred with respect to ${\mathcal {D}}_2$

The following definition is illustrated in Figs. 8, 9 and 10 .

### Definition 11

Let ${\mathcal {D}}_i$ in $\{{\mathcal {D}}_1, {\mathcal {D}}_2, {\mathcal {D}}_3\}$ be a disk that is 1-intersecting, say ${\mathcal {D}}_i={\mathcal {D}}_2$. We denote by $\tau _2$ the external tangent of ${\mathcal {D}}_1$ and ${\mathcal {D}}_3$ that ${\mathcal {D}}_2$ intersects. We denote by $A_2$ the part of ${\mathcal {D}}_2$ that is on the same side of $\tau _2$ as ${\mathcal {D}}_1$ and ${\mathcal {D}}_3$. Let ${\mathcal {D}}'$ be a disk intersecting pairwise with ${\mathcal {D}}_1$, ${\mathcal {D}}_2$ and ${\mathcal {D}}_3$. We say that ${\mathcal {D}}'$ is *outside-containing* ${\mathcal {D}}_2$ if ${\mathcal {D}}_2 \setminus A_2$ is a subset of ${\mathcal {D}}'$. We denote by $\chi _1$ and $\chi _2$ the points where the boundaries of ${\mathcal {D}}'$ and ${\mathcal {D}}_2$ intersect. Note that they are both in $A_2$. We denote by ${\mathcal {H}}$ the closed halfplane with bounding line $(\chi _1,\chi _2)$ that contains ${\mathcal {D}}_2 \setminus A_2$. Let ${\mathcal {H}}'$ be the closed halfplane with bounding line $\tau _2$ that contains $A_2$. Note that $({\mathcal {H}}\cap {\mathcal {H}}') \setminus A_2$ is the union of one or two connected sets. We have ${\mathcal {D}}' \cap {\mathcal {D}}_1 \subset ({\mathcal {H}}\cap {\mathcal {H}}') \setminus A_2$ and ${\mathcal {D}}' \cap {\mathcal {D}}_3 \subset ({\mathcal {H}}\cap {\mathcal {H}}') \setminus A_2$. If ${\mathcal {D}}' \cap {\mathcal {D}}_1$ and ${\mathcal {D}}' \cap {\mathcal {D}}_3$ are not in the same connected set, we say that ${\mathcal {D}}'$ is *centred with respect to* ${\mathcal {D}}_2$.

Let us consider a disk in $\{{\mathcal {D}}_1,{\mathcal {D}}_2,{\mathcal {D}}_3\}$, say ${\mathcal {D}}_2$, and let us assume it is the disk in the middle of a line transversal. Let us denote by $\tau$ and $\tau '$ the two external tangents of ${\mathcal {D}}_1$ and ${\mathcal {D}}_3$. Let ${\mathcal {D}}$ be a disk in $\{ {\mathcal {D}}'_j\}$, such that there exists a pair of points $p_1 \in {\mathcal {D}}\cap {\mathcal {D}}_1$ and $p_3 \in {\mathcal {D}}\cap {\mathcal {D}}_3$, such that the segment $[p_1,p_3]$ intersects ${\mathcal {D}}_2$, potentially at a single point. Without loss of generality, we can even assume that $[p_1,p_3]\cap {\mathcal {D}}_1 = \{p_1\}$ and $[p_1,p_3]\cap {\mathcal {D}}_3 = \{p_3\}$. The segment $[p_1,p_3]$ splits ${\mathcal {D}}_2$ into two closed parts. One them can potentially be a single point *p* if $[p_1,p_3]\cap {\mathcal {D}}_2=\{p\}$. Observe that ${\mathcal {D}}$ contains exactly one of those two parts: at least one because of the pseudo-disk property, and at most one because ${\mathcal {D}}$ does not contain ${\mathcal {D}}_2$. Let us denote by $A_2\subset {\mathcal {D}}_2$ the part that is contained in ${\mathcal {D}}$. We say that the side of $[p_1,p_3]$ where $A_2$ lies is the *positive side* of $[p_1,p_3]$. By definition, $p_1$ and $p_3$ lie between $\tau$ and $\tau '$. By moving $p_1$ and $p_3$ toward the positive side of $[p_1,p_3]$ and along the boundary of ${\mathcal {D}}_1$ and ${\mathcal {D}}_3$ respectively, only two things can happen by construction: Either both of them reach $\tau$, or both of them reach $\tau '$.

### Definition 12

We denote by $X_{{\mathcal {D}}_2}$ (or simply by *X* when there is no risk of confusion) the set of disks in $\{ {\mathcal {D}}'_j\}$ for which the external tangent reached is $\tau$. Likewise, we denote by $X'_{{\mathcal {D}}_2}$ (or simply by $X'$) the set of disks in $\{ {\mathcal {D}}'_j\}$ for which the external tangent reached is $\tau '$.

Let us now assume the existence of a disk ${\mathcal {D}}' \in \{ {\mathcal {D}}'_j\}$, which is not in $X \cup X'$. Let us consider $p_1 \in {\mathcal {D}}' \cap {\mathcal {D}}_1$ and $p_3 \in {\mathcal {D}}' \cap {\mathcal {D}}_3$. By assumption, the segment $[p_1,p_3]$ does not intersect ${\mathcal {D}}_2$. Observe that this implies that ${\mathcal {D}}_2$ is not 2-intersecting. It is possible to continuously move $p_1$ and $p_3$ in ${\mathcal {D}}_1$ and ${\mathcal {D}}_3$ respectively, such that they both reach either $\tau$ or $\tau '$, and while maintaining the property that $[p_1,p_3]\cap {\mathcal {D}}_2=\emptyset$. Observe that the choice of $p_1\in {\mathcal {D}}\cap {\mathcal {D}}_1$ and $p_3\in {\mathcal {D}}\cap {\mathcal {D}}_3$ has no impact on whether they can both reach $\tau$, or both reach $\tau '$. Otherwise, it would be possible to move them from $\tau$ to $\tau '$ without having $[p_1,p_3]$ intersecting ${\mathcal {D}}_2$, which would imply that ${\mathcal {D}}_2$ is not the disk in the middle of any line transversal. If ${\mathcal {D}}_2$ is 1-intersecting, then exactly one of $\tau ,\tau '$ will always be reached, for any disk in $\{ {\mathcal {D}}'_j\}$.

### Definition 13

We denote by $Y_{{\mathcal {D}}_2}$ (or simply by *Y* when there is no risk of confusion) the set of disks in $\{ {\mathcal {D}}'_j\}\setminus (X_{{\mathcal {D}}_2}\cup X'_{{\mathcal {D}}_2})$ for which the external tangent reached is $\tau$, and which are not centred with respect to ${\mathcal {D}}_1$ or ${\mathcal {D}}_3$. Likewise, we denote by $Y'_{{\mathcal {D}}_2}$ (or simply by $Y'$) the set of disks for which the external tangent reached is $\tau '$, and which are not centred with respect to ${\mathcal {D}}_1$ or ${\mathcal {D}}_3$. We denote by $Z_{{\mathcal {D}}_2}$ (or simply by *Z*), the set of disks for which the external tangent reached is $\tau '$, and which are centred with respect to ${\mathcal {D}}_1$ or ${\mathcal {D}}_3$. Finally, we denote by $Z'_{{\mathcal {D}}_2}$ (or simply by $Z'$), the set of disks for which the external tangent reached is $\tau$, and which are centred with respect to ${\mathcal {D}}_1$ or ${\mathcal {D}}_3$.

We want to emphasise the fact that indeed in the definition of *Z*, $p_1$ and $p_3$ can reach $\tau '$ and not $\tau$. This choice of notation comes from the fact that, assuming that ${\mathcal {D}}_1$ and ${\mathcal {D}}_3$ are the disks in the middle of no line transversal, or that they are 1-intersecting, all pairs of disks in $X\cup Y \cup Z$ intersect, and the same holds with all pairs of disks in $X'\cup Y' \cup Z'$, as we show later. Observe that if a disk is centred with respect to ${\mathcal {D}}_1$, then ${\mathcal {D}}_1$ is 1-intersecting. Thus if both ${\mathcal {D}}_1$ and ${\mathcal {D}}_3$ are not 1-intersecting, the sets *Z* and $Z'$ are empty.

### Lemma 12

If ${\mathcal {D}}$ and ${\mathcal {D}}'$ are in $X_{{\mathcal {D}}_2}$, then they intersect.

### Proof

Let $p_1,p_3,p'_1,p'_3$ be points coming from the definition of ${\mathcal {D}}$ and ${\mathcal {D}}'$ being in *X*. Recall that $p_1$ and $p'_1$ lie on the boundary of ${\mathcal {D}}_1$. Similarly, $p_3$ and $p'_3$ lie on the boundary of ${\mathcal {D}}_3$. If $[p_1,p_3]$ and $[p'_1,p'_3]$ intersect then we are done. Otherwise, we can assume without loss of generality that $p_1$ is closer to $\tau$ than $p'_1$ is, and that $p_3$ is closer to $\tau$ than $p'_3$ is (when considering them as points on the boundaries of ${\mathcal {D}}_1$ and ${\mathcal {D}}_3$ respectively). Let $A_2$, respectively $A'_2$, be the part of ${\mathcal {D}}_2$ contained in ${\mathcal {D}}$, respectively in ${\mathcal {D}}'$. By assumption, $A_2$ is contained in $A'_2$, which implies that the two disks intersect. $\square$

### Lemma 13

Let us assume that ${\mathcal {D}}_1$ is the disk in the middle of no line transversal or is 1-intersecting, and that the same holds with ${\mathcal {D}}_3$. If ${\mathcal {D}}$ and ${\mathcal {D}}'$ are in $Y_{{\mathcal {D}}_2}$, then they intersect.

**Fig. 11:**
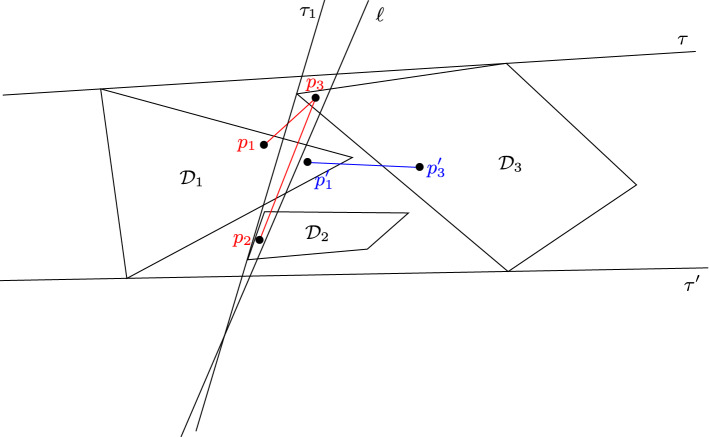
Illustration of Lemma 13. The segment $[p_2,p_3]$ splits ${\mathcal {D}}_1$, thus ${\mathcal {D}}$ contains $p'_1$ or is centred with respect to ${\mathcal {D}}_1$

### Proof

The proof is illustrated in Fig. 11. Let $p_1,p_3,p'_1,p'_3$ be points in ${\mathcal {D}}\cap {\mathcal {D}}_1$, ${\mathcal {D}}\cap {\mathcal {D}}_3$, ${\mathcal {D}}' \cap {\mathcal {D}}_1$ and ${\mathcal {D}}' \cap {\mathcal {D}}_3$, respectively. Let us assume for a contradiction that ${\mathcal {D}}$ and ${\mathcal {D}}'$ do not intersect. Let $p_2$ be in ${\mathcal {D}}\cap {\mathcal {D}}_2$ and $p'_2$ be in ${\mathcal {D}}' \cap {\mathcal {D}}_2$. We claim that $p_2$ is not in the quadrilateral $p_1p_3p'_3p'_1$. By assumption, it is possible to continuously move the points $p_1,p_3$ in ${\mathcal {D}}_1$ and ${\mathcal {D}}_3$ respectively such that $p_1$ and $p'_1$ overlap, the points $p_3$ and $p'_3$ overlap, while keeping the property that $[p_1,p_3]$ does not intersect ${\mathcal {D}}_2$. Observe that this is not possible if $p_2$ is in the quadrilateral $p_1p_3p'_3p'_1$, because ${\mathcal {D}}_1$ and ${\mathcal {D}}_3$ do not intersect with ${\mathcal {D}}_2$. Moreover by assumption ${\mathcal {D}}_2$ does not intersect with the segments $[p_1,p_3]$, $[p_3,p'_3]$, $[p'_3,p'_1]$ and $[p'_1,p_1]$. This implies that ${\mathcal {D}}_2$ is outside of the quadrilateral $p_1p_3p'_3p'_1$. Let us consider a separating line $\ell$ of ${\mathcal {D}}$ and ${\mathcal {D}}'$. As $\ell$ intersects the segments $[p_1,p'_1]$, $[p_2,p'_2]$ and $[p_3,p'_3]$, we observe that $\ell$ is a line transversal of $\{{\mathcal {D}}_1,{\mathcal {D}}_2,{\mathcal {D}}_3\}$. Let us consider the intersection *s* of $\ell$ with the quadrilateral $p_1p_3p'_3p'_1$. This intersection *s* could a priori be the union of two segments if the quadrilateral $p_1p_3p'_3p'_1$ is not convex, however that is not possible since $\ell$ does not intersect $[p_1,p_3]$ and $[p'_1,p'_3]$. We have that *s* is a segment with one endpoint in ${\mathcal {D}}_1$ and the other in ${\mathcal {D}}_3$. This implies that the disk in the middle of $\ell$ is not ${\mathcal {D}}_2$.

If ${\mathcal {D}}_2$ is the disk in the middle of all line transversals, we have already reached a contradiction. Let us now assume that at least one of ${\mathcal {D}}_1$ and ${\mathcal {D}}_3$ is 1-intersecting. Without loss of generality, let us assume that ${\mathcal {D}}_1$ is the disk in the middle of $\ell$. We denote by $\tau _1$ the external tangent of ${\mathcal {D}}_2$ and ${\mathcal {D}}_3$ which intersects ${\mathcal {D}}_1$. Both segments $[p_2,p_3]\cap {\mathcal {D}}_1$ and $[p'_2,p'_3] \cap {\mathcal {D}}_1$ are on the same side of $\tau _1$. By assumption, only one of the two segments can be continuously moved to $\tau _1$ without touching $\ell$. Without loss of generality, let us assume that this segment is $[p_2,p_3]\cap {\mathcal {D}}_1$. Observe that $[p_2,p_3]$ and $p'_1$ are on different sides of $\ell$. The line segment $[p_2,p_3]$ splits ${\mathcal {D}}_1$ into two parts, one of them being contained in ${\mathcal {D}}$. This implies that ${\mathcal {D}}$ contains $p'_1$ or is centred with respect to ${\mathcal {D}}_1$, which is a contradiction. $\square$

### Lemma 14

Let us assume that ${\mathcal {D}}_1$ is the disk in the middle of no line transversal or is 1-intersecting, and that the same holds with ${\mathcal {D}}_3$. If ${\mathcal {D}}$ is in $X_{{\mathcal {D}}_2}$ and ${\mathcal {D}}'$ is in $Y_{{\mathcal {D}}_2}$, then they intersect.

### Proof

Let $p_1,p_3$ be points coming from the definition of ${\mathcal {D}}$ being in *X*. Let $p'_1,p'_3$ be points in ${\mathcal {D}}' \cap {\mathcal {D}}_1$ and ${\mathcal {D}}' \cap {\mathcal {D}}_3$, respectively. By definition, the segment $[p_1,p_3]$ splits ${\mathcal {D}}_2$ into two parts. We denote by $A_2$ the part of ${\mathcal {D}}_2$ that is contained in ${\mathcal {D}}$. Assume for a contradiction that ${\mathcal {D}}$ and ${\mathcal {D}}'$ do not intersect. We claim that $A_2$ is inside the quadrilateral $p_1p_3p'_3p'_1$. Without loss of generality, let us assume that $[p'_1,p'_3]\cap {\mathcal {D}}_1 = \{p'_1\}$ and $[p'_1,p'_3]\cap {\mathcal {D}}_3 = \{p'_3\}$. It is possible to move $p'_1$ and $p'_3$ to $\tau$, while following the boundaries of ${\mathcal {D}}_1$ and ${\mathcal {D}}_3$ respectively, such that $[p'_1,p'_3]$ does not intersect ${\mathcal {D}}_2$. Since ${\mathcal {D}}$ and ${\mathcal {D}}'$ do not intersect, it implies that $[p_1,p_3]$ and $[p'_1,p'_3]$ do not intersect. In particular, it implies that $p'_1$ is closer to $\tau$ than $p_1$ (when considering them as points on the boundary of ${\mathcal {D}}_1$), and likewise $p'_3$ is closer to $\tau$ than $p_3$ is. By assumption, ${\mathcal {D}}_2$ does not intersect the segments $[p_1,p'_1]$, $[p'_1,p'_3]$ and $[p'_3,p_3]$. We have shown that $A_2$ is inside the quadrilateral $p_1p_3p'_3p'_1$.

As ${\mathcal {D}}$ and ${\mathcal {D}}'$ are not intersecting, we have that ${\mathcal {D}}'\cap A_2$ is empty. Let us denote by $\ell$ a separating line of ${\mathcal {D}}$ and ${\mathcal {D}}'$. As $\ell$ does not intersect $[p_1,p_3]$ or $[p'_1,p'_3]$, but because $\ell$ intersects $[p_1,p'_1]$ and $[p_3,p'_3]$, we have that $\ell$ splits the quadrilateral $p_1p_3p'_3p'_1$. Furthermore, $\ell$ does not intersect $A_2$, and thus it is a line transversal of $\{{\mathcal {D}}_1,{\mathcal {D}}_2,{\mathcal {D}}_3\}$ whose disk in the middle is not ${\mathcal {D}}_2$. If ${\mathcal {D}}_2$ is the disk in the middle of all line transversals, we have already reached a contradiction. Let us now assume that at least one of ${\mathcal {D}}_1$ and ${\mathcal {D}}_3$ is 1-intersecting. Without loss of generality, let us assume that the disk in the middle of $\ell$ is ${\mathcal {D}}_1$. Let $\tau _1$ be the external tangent of ${\mathcal {D}}_2$ and ${\mathcal {D}}_3$ which intersects ${\mathcal {D}}_1$. Let $p'_2$ be in ${\mathcal {D}}' \cap D_2$. Now, the segment $[p'_2,p'_3]\cap {\mathcal {D}}_1$ lies between the lines $\tau _1$ and $\ell$, and $[p'_2,p'_3]$ splits ${\mathcal {D}}_1$ in such a way that ${\mathcal {D}}'$ either contains $p_1$ or is centred with respect to ${\mathcal {D}}_1$, which is a contradiction. $\square$

**Fig. 12:**
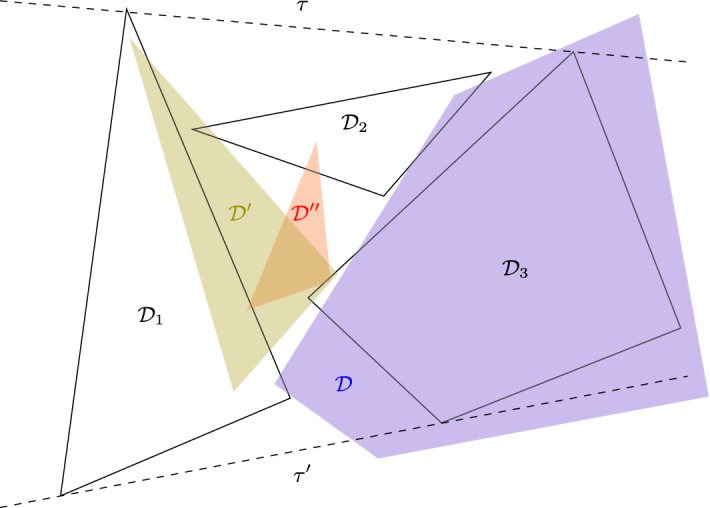
The disk ${\mathcal {D}}$ is in *Z*, the disk ${\mathcal {D}}'$ is in $X'$ and the disk ${\mathcal {D}}''$ is in $Y'$. Observe that ${\mathcal {D}}$ does not intersect ${\mathcal {D}}'$ or ${\mathcal {D}}''$

We have now shown that under certain conditions, $G(X\cup Y)$ and $G(X' \cup Y')$ are complete graphs. We now prove three lemmas to show that $G(X\cup Y\cup Z)$ and $G(X' \cup Y'\cup Z')$ are complete graphs. To do so, we have to show that all pairs of disks in *Z* intersect. In the following lemma, we prove the stronger statement that all pairs of disks in $Z\cup Z'$ intersect. Then, in Lemmas 16 and 17 , we show that a disk ${\mathcal {D}}$ in *Z* intersects any disk in *X* or *Y*. This implies that if ${\mathcal {D}}$ does not intersect a disk ${\mathcal {D}}'$ in $\{ {\mathcal {D}}'_j\}$, then ${\mathcal {D}}'$ is in $X'\cup Y'$, as illustrated in Fig. 12.

### Lemma 15

Let ${\mathcal {D}}'$ and ${\mathcal {D}}''$ be intersecting with $\{{\mathcal {D}}_1, {\mathcal {D}}_2,{\mathcal {D}}_3\}$. If ${\mathcal {D}}'$ and ${\mathcal {D}}''$ are respectively centred with respect to ${\mathcal {D}}_i$ and ${\mathcal {D}}_j$, $i,j \in \{1,2,3\}$, then they intersect.

**Fig. 13:**
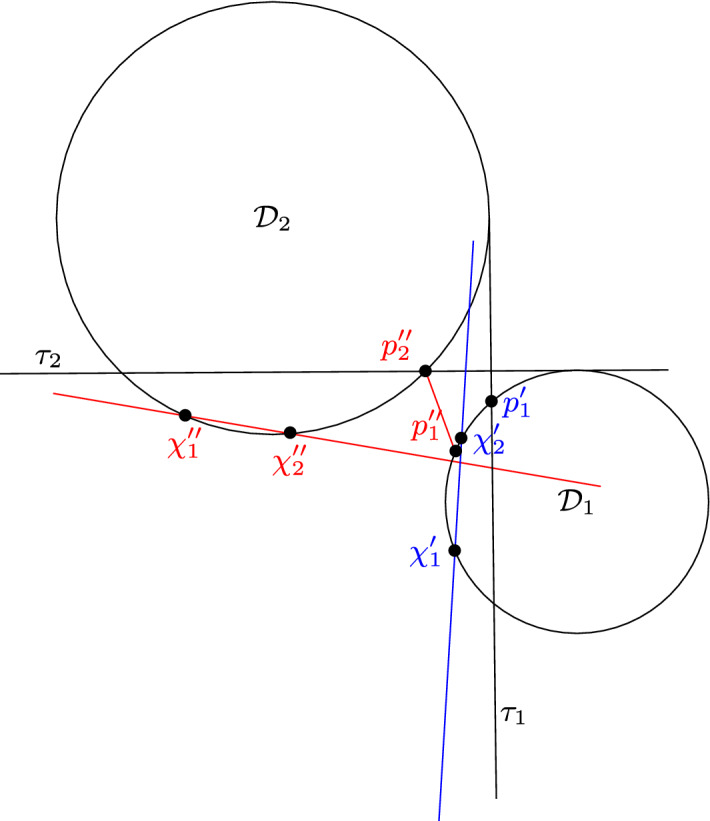
Illustration of the proof of Lemma 15

### Proof

The proof is illustrated in Fig. 13. For a disk ${\mathcal {D}}_i$ in $\{{\mathcal {D}}_1, {\mathcal {D}}_2, {\mathcal {D}}_3\}$ which is 1-intersecting, we denote by $\tau _i$ the external tangent of the two other disks that ${\mathcal {D}}_i$ intersects. Furthermore, we denote by $A_i$ the subset of ${\mathcal {D}}_i$ which lies on the same side of $\tau _i$ as the two other disks. Let ${\mathcal {D}}'$ and ${\mathcal {D}}''$ be two disks in that are centred. If they both contain the same subset ${\mathcal {D}}_i \setminus A_i$ for some $i \in \{1,2,3\}$, then they intersect. Otherwise, let us assume without loss of generality that ${\mathcal {D}}'$ is centred with respect to ${\mathcal {D}}_1$ and ${\mathcal {D}}''$ is centred with respect to ${\mathcal {D}}_2$. There are two intersections between the boundaries of ${\mathcal {D}}''$ and ${\mathcal {D}}_2$, that we denote by $\chi ''_1$ and $\chi ''_2$. We denote by ${\mathcal {H}}''$ the closed halfplane with bounding line $(\chi ''_1,\chi ''_2)$ that contains ${\mathcal {D}}_2 \setminus A_2$. We denote by ${\mathcal {H}}_2$ the closed halfplane with bounding line $\tau _2$ that contains $A_2$. By assumption, ${\mathcal {D}}_1$ intersects only one of the two connected sets of $({\mathcal {H}}'' \cap {\mathcal {H}}_2) \setminus A_2$. Let us consider the intersections of $\tau _2$ with the boundary of ${\mathcal {D}}_2$. By what we just said, there is a closest intersection to ${\mathcal {D}}_1$, that we denote by $p''_2$. Note that $p''_2$ is in ${\mathcal {H}}''$, and therefore in ${\mathcal {D}}''$. Let $p''_1$ be a point in ${\mathcal {D}}'' \cap D_1$. If $p''_1$ is in ${\mathcal {D}}'$ then we are done. Let us now assume that it is not the case. We denote by $\chi '_1$ and $\chi '_2$ the intersections of ${\mathcal {D}}'$ with the boundary of ${\mathcal {D}}_1$. Without loss of generality, we can assume that $p''_1$ is on the boundary of ${\mathcal {D}}_1$. We denote by $p'_1$ the intersection of $\tau _1$ and the boundary of ${\mathcal {D}}_1$ that is the closest to ${\mathcal {D}}_2$, which can be defined similarly to how we defined $p''_2$. Now observe that one of $\chi '_1$ and $\chi '_2$ is between $p''_1$ and $p'_1$ on the boundary of ${\mathcal {D}}_1$. Assume without loss of generality that $\chi '_2$ is the closest to ${\mathcal {D}}_2$. Let us consider the halfplane ${\mathcal {H}}'$ with bounding line $(\chi '_1,\chi '_2)$ that contains ${\mathcal {D}}_1 \setminus A_1$. We also denote by ${\mathcal {H}}_1$ the halfplane with bounding line $\tau _1$ that contains $A_1$. As ${\mathcal {D}}'$ is centred with respect to ${\mathcal {D}}_1$, there is one of the two connected component that intersects with ${\mathcal {D}}_2$, and the other with ${\mathcal {D}}_3$. Note that the connected component on the side of $\chi '_2$ cannot intersect with ${\mathcal {D}}_3$, since otherwise ${\mathcal {D}}_1$ would not be the disk in the middle of $\tau _1$. This implies that the connected component on the side of $\chi '_2$ is the one that intersects ${\mathcal {D}}_2$. Finally, observe that either ${\mathcal {D}}'$ contains $p''_2$, or it does not intersect with $A_2$, and thus contains a point in ${\mathcal {D}}_2 \setminus A_2$. In both cases, ${\mathcal {D}}'$ contains a point in ${\mathcal {D}}''$. $\square$

### Lemma 16

Let us assume that ${\mathcal {D}}_1$ or ${\mathcal {D}}_3$ is 1-intersecting. If ${\mathcal {D}}$ is in $X_{{\mathcal {D}}_2}$ and ${\mathcal {D}}'$ is in $Z_{{\mathcal {D}}_2}$, then they intersect.

### Proof

Let $p_1,p_3$ be the points coming from the definition of ${\mathcal {D}}$ being in *X*. By assumption, the segment $[p_1,p_3]$ splits ${\mathcal {D}}_2$ into two parts. Let us denote by $A_2$ the part that is contained in ${\mathcal {D}}$. Without loss of generality, let us assume that ${\mathcal {D}}'$ is centred with respect to ${\mathcal {D}}_1$. We have that $p_1$ is on the boundary of ${\mathcal {D}}_1$. Let us denote by $p'_1$ and $q'_1$ the two intersections between the boundaries of ${\mathcal {D}}'$ and ${\mathcal {D}}_1$. Let us consider the boundary of ${\mathcal {D}}_1$. The lines $\tau$ and $\tau '$ cut it into two parts, one of them containing $p_1$, $p'_1$ and $q'_1$. Let us consider that part of the boundary of ${\mathcal {D}}_1$. We assume that when going from $\tau$ to $\tau '$ while following this part, we see the points in that order: $p'_1$, $p_1$ and then $q'_1$. Indeed if $p_1$ does not appear between $p'_1$ and $q'_1$, then $p_1$ is in ${\mathcal {D}}'$ and we are done. We can assume without loss of generality that we reach first $p'_1$ and last $q'_1$ by relabelling if need be. Let us follow the boundary of ${\mathcal {D}}'$ from $p'_1$ while staying outside of ${\mathcal {D}}_1$ (therefore not going in the direction of $q'_1$), until we reach either ${\mathcal {D}}_2$ or ${\mathcal {D}}_3$. This must happen as ${\mathcal {D}}'$ is centred with respect to ${\mathcal {D}}_1$. We claim that we can only reach ${\mathcal {D}}_2$. Indeed, assume for a contradiction that we reach a point $p'_3$ at an intersection between the boundaries of ${\mathcal {D}}'$ and ${\mathcal {D}}_3$. As ${\mathcal {D}}'$ is in *Z*, the segment $[p'_1,p'_3]$ does not intersect ${\mathcal {D}}_2$. However, the segment $[p_1,p_3]$ does intersect ${\mathcal {D}}_2$. As $[p_1,p_3]$ and $[p'_1,p'_3]$ do not intersect, and since $p'_1$ is closer to $\tau$ than $p_1$ is (when considering their positions on the boundary of ${\mathcal {D}}_1$), it implies that $p'_3$ is closer to $\tau$ than $p_3$ is (when considering their positions on the boundary of ${\mathcal {D}}_3$). It is now possible to continuously move $p'_1$ and $p'_3$ to $\tau$ while keeping the property that $[p'_1,p'_3]$ does not intersect ${\mathcal {D}}_2$, which is impossible. We have shown that when following the boundary of ${\mathcal {D}}'$ from $p'_1$, we meet a point $p'_2$ in ${\mathcal {D}}' \cap {\mathcal {D}}_2$. By construction, as $p'_1$ is closer to $\tau$ than $p_1$ is, we know that $p'_2$ is in $A_2$, which implies that ${\mathcal {D}}$ and ${\mathcal {D}}'$ intersect. $\square$

### Lemma 17

Let us assume that ${\mathcal {D}}_1$ or ${\mathcal {D}}_3$ is 1-intersecting. If ${\mathcal {D}}$ is in $Y_{{\mathcal {D}}_2}$ and ${\mathcal {D}}'$ is in $Z_{{\mathcal {D}}_2}$, then they intersect.

### Proof

Assume for a contradiction that they do not intersect. Let $p_1$ and $p_3$ be in ${\mathcal {D}}\cap {\mathcal {D}}_1$ and ${\mathcal {D}}\cap {\mathcal {D}}_3$ respectively. We can even assume that $[p_1,p_3]\cap {\mathcal {D}}_1=\{p_1\}$ and $[p_1,p_3]\cap {\mathcal {D}}_3=\{p_3\}$. Without loss of generality, let us assume that ${\mathcal {D}}'$ is centred with respect to ${\mathcal {D}}_1$. We define $p'_1$ and $q'_1$ as in the proof of Lemma 16. We follow the boundary of ${\mathcal {D}}'$ from $p'_1$ while staying outside of ${\mathcal {D}}_1$ (therefore not going in the direction of $q'_1$), until we reach either ${\mathcal {D}}_2$ or ${\mathcal {D}}_3$. Again, this must happen as ${\mathcal {D}}'$ is centred with respect to ${\mathcal {D}}_1$. However, we cannot reach ${\mathcal {D}}_3$ because if so, by denoting $p'_3$ the point on the boundary of ${\mathcal {D}}_3$ that we reach, the points $p'_1$ and $p'_3$ could be moved to $\tau$ while having $[p'_1,p'_3]$ not intersecting ${\mathcal {D}}_2$. This is because $p'_1$ is closer to $\tau$ than $p_1$ is (when considering their positions on the boundary of ${\mathcal {D}}_1$), and the same holds with $p'_3$ and $p_3$. Since ${\mathcal {D}}$ is in *Y*, $[p_1,p_3]$ can be moved to $\tau$ without intersecting ${\mathcal {D}}_2$, and thus so can $[p'_1,p'_3]$. This implies that the disk we reach is ${\mathcal {D}}_2$. But this is in contradiction with the fact that $[p_1,p_3]$ can be moved to $\tau$ without intersecting ${\mathcal {D}}_2$, since $p'_1$ is closer to $\tau$ than $p_1$ is. $\square$

We can now prove Theorem 4.

### Proof of Theorem 4

We consider any fixed representation of *G* with convex pseudo-disks. We denote by ${\mathcal {D}}_1$, ${\mathcal {D}}_2$ and ${\mathcal {D}}_3$ the three non-intersecting sets corresponding to *H*. Likewise we denote by $\{ {\mathcal {D}}'_j\}$ the convex pseudo-disks in $G\setminus H$. If there is no line transversal of $\{ {\mathcal {D}}_1,{\mathcal {D}}_2, {\mathcal {D}}_3\}$, we conclude with Lemma 9. If there is one disk that is 2-intersecting, say ${\mathcal {D}}_2$, then we have that *Y*, $Y'$, *Z* and $Z'$ are empty. We conclude with Lemma 12. Now let us assume that one disk, say ${\mathcal {D}}_2$ is contained. Then we know with Lemma 11 that ${\mathcal {D}}_1$ is the disk in the middle of no line transversal, or it 1-intersecting. The same holds with ${\mathcal {D}}_3$. Therefore we can apply Lemmas 12, 13, 14, 15, 16 and 17 . They imply that $G(X\cup Y \cup Z)$ is a complete graph. By the same arguments, $G(X'\cup Y' \cup Z')$ is a complete graph too. As $\{ {\mathcal {D}}'_j\}$ is the disjoint union of *X*, $X'$, *Y*, $Y'$, *Z* and $Z'$, it implies that $G\setminus H$ is cobipartite. If no disk is 2-intersecting and no disk is contained, then all disks are either the disk in the middle of no line transversal, or are 1-intersecting. As we are now assuming that there is a line transversal of $\{ {\mathcal {D}}_1,{\mathcal {D}}_2, {\mathcal {D}}_3\}$, we assume without loss of generality that its disk in the middle is ${\mathcal {D}}_2$, and we use Lemmas 12, 13, 14, 15, 16 and 17 to conclude. $\square$

## Proof of Theorem 3

We first give some definitions. Vapnik and Chervonenkis have introduced the concept of VC-dimension in Vapnik and Chervonenkis (1971). In this paper, we are only concerned with the VC-dimension of the neighbourhood of some geometric intersection graphs. In this context, the definition can be stated as follows:

### Definition 14

Let ${\mathcal {F}}$ be a family of sets in ${\mathbb {R}}^d$, and let *G* be the intersection graph of ${\mathcal {F}}$. We say that $F\subseteq {\mathcal {F}}$ is *shattered* if for every subset *X* of *F*, there exists a vertex *v* in *G* that is adjacent to all vertices in *X*, and adjacent to no vertex in $F\setminus X$. The VC-dimension of the neighbourhood of *G* is the maximum cardinality of a shattered subset of ${\mathcal {F}}$.

We define the class ${\mathcal {X}}(d,\beta ,K)$ as introduced by Bonamy et al. (2018). Let *d* and *K* be in ${\mathbb {N}}$, and let $\beta$ be a real number such that $0<\beta \le 1$. Then ${\mathcal {X}}(d,\beta ,K)$ denotes the class of simple graphs *G* such that the VC-dimension of the neighbourhood of *G* is at most *d*, $\alpha (G)\ge \beta |V(G)|$, and $\text {iocp}(G)\le K$. They show that there exist EPTAS (Efficient Polynomial-Time Approximation Scheme) for computing a maximum independent set in ${\mathcal {X}}(d,\beta ,K)$. An EPTAS for a maximisation problem is an approximation algorithm that takes a parameter $\varepsilon >0$ and outputs a $(1-\varepsilon )$-approximation of an optimal solution, and running in $f(\varepsilon )n^{{\mathcal {O}}(1)}$ time. More formally, we have the following:

### Theorem 6

(Bonamy et al. 2018) For any constants $d,K \in {\mathbb {N}}$, $0<\beta \le 1$, for every $\varepsilon > 0$, there is a randomised $(1-\varepsilon )$-approximation algorithm running in time $2^{\tilde{{\mathcal {O}}}(1/\varepsilon ^3)}n^{{\mathcal {O}}(1)}$ for maximum independent set on graphs of ${\mathcal {X}}(d,\beta ,K)$ with *n* vertices.

Recently, Dvořák and Pekárek (2020) have announced that it is not necessary to have bounded VC-dimension. More explicitly, there is an EPTAS for the class ${\mathcal {X}}(+\infty ,\beta ,K)$. However, their running time dependence in *n* is higher: $\tilde{{\mathcal {O}}}(n^5)$ with Dvořák and Pekárek’s algorithm compared to $\tilde{{\mathcal {O}}}(n^2)$ with the one of Bonamy *et al.* Also, Dvořák and Pekárek do not compute the dependence in $\varepsilon$. For this reason, we prefer the algorithm of Bonamy *et al.*, despite the fact that we have to show bounded VC-dimension.

Theorem 6 states that there exists an EPTAS for computing a maximum independent set on graphs of ${\mathcal {X}}(d,\beta ,K)$, for any $d,K \in {\mathbb {N}}$ and $0<\beta \le 1$. Let *G* be in $\varPi ^3$. In order to prove Theorem 3, we show that the VC-dimension of the neighbourhood of any vertex in *G* is bounded. Observe that the VC-dimension of a graph and its complement are equal. We aim at using the EPTAS mentioned above for computing a maximum independent set in the complement, which is equivalent to computing a maximum clique in the original graph. However a graph *G* in $\varPi ^3$ does not necessarily satisfy $\alpha ({\overline{G}}) \ge \beta |V(G)|$ for some $0< \beta \le 1$. Even if it does, we need to know the value of $\beta$ in order to use the EPTAS of Theorem 6. Therefore we show how to compute a maximum clique in any $G\in \varPi ^3$ by using polynomially many times the EPTAS of Theorem 6 on some subgraphs of *G*, which have the desired property.

In general, for intersection graphs of geometric objects that can be described with finitely many parameters, the VC-dimension of the neighbourhood is bounded. For graphs in $\varPi ^3$, we were able to show an upper bound of 28. We do not expect this value to be tight, but showing any constant was sufficient for our purpose.

### Proposition 1

The VC-dimension of the neighbourhood of a graph $G=(V,E)$ in $\varPi ^3$ is at most 28.

We use the fact that the VC-dimension of the neighbourhood of disk graphs (and even pseudo-disk graphs) is at most 4, as proved by Aronov et al. (2018). Likewise, the VC-dimension of the neighbourhood of unit ball graphs is at most 4, as noticed by Bonamy et al. (2018). For any point $c\in {\mathbb {R}}^3$ and any non-negative real number $\rho$, we denote by ${\mathcal {B}}(c,\rho )$ the ball centred at *c* with radius $\rho$. Moreover, we denote by $P^3(c,\rho )$ the 3-pancake that is the Minkowski sum of the unit ball centred at the origin and the disk lying on the plane *xOy*, centred at *c* with radius $\rho$. Note that if $\rho =0$, then $P^3(c,\rho )$ is the unit ball centred at *c*. Before showing Proposition 1, we show the following:

### Lemma 18

Let ${\mathcal {B}}$ be a unit ball centred at *c* and let $P^3(c',\rho )$ be a 3-pancake. We denote by ${\mathcal {D}}$ the disk that is the intersection of ${\mathcal {B}}(c,2)$ and the plane *xOy*. Also, we denote by ${\mathcal {D}}'$ the disk ${\mathcal {D}}(c',\rho )$ (which is a strict subset of the intersection of $P^3$ and the plane *xOy*). We have that ${\mathcal {B}}$ and $P^3$ intersect if and only if ${\mathcal {D}}$ and ${\mathcal {D}}'$ intersect.

### Proof

By definition, ${\mathcal {B}}$ and $P^3$ intersect if and only if there exists a unit ball ${\mathcal {B}}'$ whose centre lies in ${\mathcal {D}}'$ such that ${\mathcal {B}}$ and ${\mathcal {B}}'$ intersect. This is equivalent to say that ${\mathcal {B}}(c,2)$ contains a point in ${\mathcal {D}}'$. Finally, this statement is equivalent to having ${\mathcal {D}}$ and ${\mathcal {D}}'$ intersecting. $\square$

### Proof of Proposition 1

First let us show that if *V* is shattered, then in any $\varPi ^3$ representation of *G* there are at most four 3-pancakes. Let us assume by contradiction that there exists a set *S* of five 3-pancakes, such that for every subset *T* of *S*, there exists a unit ball or a 3-pancake intersecting all elements in *T* and intersecting no element in $S\setminus T$. For each 3-pancake $P^3(c_i,\rho _i)$ in *S*, we denote by ${\mathcal {D}}_i$ the disk ${\mathcal {D}}(c_i,\rho _i)$ lying on the plane *xOy*. Let *T* be a subset of *S*. If there exists a 3-pancake $P^3(c',\rho ')$ intersecting with the elements of *T* and with no element in $S \setminus T$, we denote by ${\mathcal {D}}_T$ the disk ${\mathcal {D}}(c',\rho '+2)$ lying on the plane *xOy*. Otherwise there exists a unit ball ${\mathcal {B}}$ centred at $c''$ intersecting intersecting with the elements of *T* and with no element in $S \setminus T$, and then we denote by ${\mathcal {D}}_T$ the intersection between ${\mathcal {B}}(c'',2)$ and *xOy*. As ${\mathcal {B}}$ intersects with a 3-pancake, ${\mathcal {D}}_T$ is not empty. Using Lemma 18, we have that ${\mathcal {D}}_i$ intersects with ${\mathcal {D}}_T$ if and only if $P^3(c_i,\rho _i)$ is in *T*. This implies that if *S* is shattered by some 3-pancakes and unit balls, then the set $\{{\mathcal {D}}_i\}$ is shattered by $\{{\mathcal {D}}_T \mid T \subseteq S\}$. However this is not possible because the VC-dimension of the neighbourhood of disk graphs is at most 4.

Now let us prove the claim. Assume by contradiction that we have a shattered set with 29 elements. As shown above, in any $\varPi ^3$ representation there are at least 25 unit balls. Let us consider such a representation. We denote by $S_1, \dots , S_5$ five sets of five unit balls each. As the VC-dimension of the neighbourhood of unit ball graphs is at most 4, for each set $S_i$ there exists a non-empty subset $T_i \subseteq S_i$ such that no unit ball can intersect with the unit balls in $T_i$, but not with those in $S_i \setminus T_i$. Therefore the absolute height of the centre of any unit ball in $T_i$ is at most 2, since $T_i$ is realised by a 3-pancake. For each $T_i$, we choose arbitrarily one unit ball ${\mathcal {B}}_i$, and define a new set *T* as $\{{\mathcal {B}}_1,\dots ,{\mathcal {B}}_5\}$. Moreover for each unit ball ${\mathcal {B}}_i$ centred at $c_i$, we denote by ${\mathcal {D}}_i$ the intersection between ${\mathcal {B}}(c_i,2)$ and the plane *xOy*. Note that ${\mathcal {D}}_i$ is not empty. Let $T'$ be a subset of *T*, and let us consider the set $\cup _{{\mathcal {B}}_i \in T'} T_i$, that we denote by $T'_+$. Note that unless $T'=\emptyset$, no unit ball can intersect with all elements in $T'_+$ and with no element in $S \setminus T'_+$. Therefore this can only be achieved by a 3-pancake $P^3(c,\rho )$, and we denote by ${\mathcal {D}}_{T'}$ the disk ${\mathcal {D}}(c,\rho )$ lying on the plane *xOy*. Using Lemma 18, the five disks ${\mathcal {D}}_i$ are shattered by the disks in $\{D_{T'}\mid T' \subseteq T\}$, which is impossible. $\square$

### Proof of Theorem 3

Let *G* be a graph in $\varPi ^3$ with *n* vertices. Since the VC-dimension of a graph is the same as its complement, Proposition 1 implies that the VC-dimension of ${\overline{G}}$ is at most 28. First let us assume that a representation of *G* is given. For every vertex represented by a unit ball, we are going to compute a maximum clique containing this vertex. As noticed by Bonamy et al. (2018), for any vertex *v* represented by a unit ball, we have $|{\mathcal {N}}(v)|\le 25 \omega (G)$. Let us denote by $G_v$ the subgraph induced by ${\mathcal {N}}(v)$. Thus we have $\alpha ({\overline{G}}_v)\ge |{\mathcal {N}}(v)|/25$. This shows that ${\overline{G}}_v$ is in ${\mathcal {X}}(28,1/25,K)$. Using Theorem 6, we have a randomised EPTAS for computing a maximum independent set in ${\overline{G}}_v$, which is equivalent to computing a maximum clique in $G_v$. Note that computing a maximum clique in $G_v$ for each vertex *v* represented by a unit ball adds at most a multiplicative factor *n* in the running time. It remains to compute a maximum clique that only contains vertices represented by 3-pancakes. Instead of considering 3-pancakes, one can only look at the corresponding disks on the plane *xOy*. This can be done as suggested in Bonamy et al. (2018): find four piercing points in time ${\mathcal {O}}(n^8)$, then consider the subgraph *H* of disks that are pierced by at least one of these points. We have $\alpha ({\overline{H}})\ge n'/4$ where $n'$ denotes the number of vertices in *H*. This implies that *H* is in ${\mathcal {X}}(28,1/4,K)$, and we can conclude as before.

Now assume that a representation is not given. As we do not know whether a vertex can be represented by a unit ball, we cannot compute a maximum clique as was done above. If there exists a representation of *G* with at least one vertex *v* represented as a unit ball, then $\alpha (G_v)\le 12$, because the kissing number for unit spheres is 12. Indeed for any 3-pancake $P^3$ intersecting a unit ball *B*, there exists a unit ball $B' \subseteq P^3$ such that *B* and $B'$ intersect. Thus, if instead of each pancake there were such a unit ball, we would have the desired inequality. But since such a unit ball $B'$ is contained in the corresponding 3-pancake $P^3$, the independence number of $G_v$ can only decrease when considering the actual 3-pancakes, which implies $\alpha (G_v)\le 12$. If there exists a representation only with 3-pancakes, then the vertex *v* corresponding the 3-pancake with the smallest radius satisfies $\alpha (G_v)\le 6$. Therefore in any case there must be a vertex *v* with $\alpha (G_v)\le 12$. We can find such a vertex in ${\mathcal {O}}(n^{13})$ time by testing for each *v* whether there is an independent of size 12 in $G_v$.

In order to give a linear lower bound on $\alpha ({\overline{G}}_v)$, we first give an upper bound on the chromatic number of any graph in $\varPi ^3$. Let ${\tilde{G}}$ be a graph in $\varPi ^3$, given with a fixed representation. We denote by $V_1$ the set of vertices represented by unit balls, and by $V_2$ those represented by 3-pancakes. We denote by ${\tilde{G}}_1$ the graph induced by $V_1$. As noted in Bonamy et al. (2018), we have for each $v_1 \in V_1$, $|{\mathcal {N}}(v_1)|\le 25 \omega ({\tilde{G}}_1)$. Since $\omega ({\tilde{G}}_1)\le \omega ({\tilde{G}})$, the maximum degree in ${\tilde{G}}_1$ is at most $25 \omega ({\tilde{G}})-1$, which implies that we can colour the vertices in $V_1$ using at most $25 \omega ({\tilde{G}})$ colours. For disk graphs, the chromatic number is at most 6 times the clique number. Thus we can colour the vertices in $V_2$ using at most $6 \omega ({\tilde{G}})$ other colours. So in total we have $\chi ({\tilde{G}})\le 31 \omega ({\tilde{G}})$.

Going back to the subgraph $G_v$, we have $\alpha (G_v) \omega (G_v) \ge \alpha (G_v) \chi (G_v)/31 \ge |{\mathcal {N}}(v)|/31$. Therefore we obtain $\omega (G_v)\ge |{\mathcal {N}}(v)|/372$. This implies that ${\overline{G}}_v$ is in ${\mathcal {X}}(28,1/372,K)$, and therefore we have an EPTAS for computing a maximum clique containing *v*. We can iterate this process in the graph *G* where *v* has been removed to compute a maximum clique that does not contain *v*. As we repeat this process linearly many times, we obtain an EPTAS for computing a maximum clique in *G*. $\square$

## Data Availability

Not applicable.
